# PFAS endocrine disruption affecting pubertal development: exposure-response is steeper below median PFAS serum concentrations

**DOI:** 10.1186/s12940-026-01285-9

**Published:** 2026-03-12

**Authors:** Nicolas van Larebeke, Stefan Voorspoels, Elly Den Hond, Martine Leermakers, Greet Schoeters

**Affiliations:** 1 Archeology, Environmental Changes and Geochemistry, Vrije Universiteit Brussel, Pleinlaan 2, Brussels, 1050 Belgium; 2 Retired from Ghent University Hospital, Study Centre for Carcinogenesis and Primary Prevention of Cancer, De Pintelaan 185, Ghent, 9000 Belgium; 3 Materials and Chemistry Unit, Flemish Institute for Technological Research (VITO), Boeretang 200, Mol, 2400 Belgium; 4 Provincial Institute of Hygiene, Kronenburgstraat 45, Antwerp, 2000 Belgium; 5 Department of Biomedical Sciences & Toxicological Centre, University of Antwerp, Universiteitsplein 1, Wilrijk, 2610 Belgium

**Keywords:** PFOS(branched), PFOA, PFHxS, PFNA, Bioavailable testosterone, FT3, Height, Growth spurt, Breast development, Low dose.

## Abstract

**Background:**

We have recently published (van Larebeke, Environ Health 24:34, 2025) consistent and biologically relevant associations of PFAS serum concentrations with sex hormone levels in male adolescents and with a significant delay in physiological processes occurring during puberty in girls and boys. Associations with thyroid hormones differed significantly by sex with only for boys significant positive associations of FT3 with PFOA, PFHxS and PFNA. Here we report a follow-up analysis to test a work hypothesis stating that, as can be expected for receptor mediated effects, the observed associations would be stronger at lower PFAS serum concentrations (e.g. below the median) than at higher PFAS serum concentrations.

**Main body:**

To allow for a comparison between associations of biological/health effects with PFAS concentrations below or above median values, PFAS concentrations without log transformation were used in linear multiple regression with continuous effect parameters, logistic models with binary effect parameters, and Ordinal Multinomial Probit models with ordinal effect parameters. Regression coefficients and other statistical parameters were calculated with the Statistica 14.0 program. Significance of the difference between a regression coefficient observed for an association with a certain PFAS below the median serum concentration compared to above the median was assessed using a two‑tailed Wald test. The median PFAS serum values observed in our study were below present health based guidance values.

Consistent with our working hypothesis, the lower exposed subpopulations (with serum concentrations below the median) had a greater change in outcome per unit increase in PFAS exposure (a stronger regression coefficient) than the higher exposed subpopulations concerning 72 of the 84 significant associations described previously and this was most pronounced for PFHxS, and to a lesser extent for PFOS(branched), PFOA and sum parameters. Continuous piecewise linear models, models with a quadratic term and, in the range of PFAS concentrations where sufficient data were present, also weighted least square graphs, yielded results supporting stronger regression coefficients below median PFAS serum concentrations.

**Conclusion:**

Our observations suggest that an important part of the endocrine disrupting effect of PFAS on adolescents occurs at serum levels below present health based guidance values.

**Supplementary Information:**

The online version contains supplementary material available at 10.1186/s12940-026-01285-9.

## Background

Per- and polyfluoroalkyl substances (PFAS) are omnipresent in the environment and in human beings and can remain a long time in the human body, with half-lives of up to 8.5 years. Health effects associated with internal exposure to PFAS include endocrine disrupting, immunotoxic and genotoxic effects, disturbance of lipid profiles, an increased risk of cancer, male infertility, diabetes and cardiovascular diseases. However, there remains uncertainty and discussion as to the PFAS serum levels that are effectively of concern.

As published in “Environmental Health” by van Larebeke et al. [1] we observed significant, consistent and biologically relevant associations of PFAS serum concentrations with sex hormone and Sex Hormone Binding Globulin (SHBG) levels in male adolescents. Moreover, we observed a significant delay in physiological processes (growth spurt, body hair development, development of pubertal skin changes, height in boys, breast development in girls, reaching a certain stage in the Pubertal Development Scale) occurring in puberty in girls and boys. Associations with thyroid hormones differed significantly by sex with only for boys significant positive associations of FT3 with PFOA, PFHxS and PFNA whereas for Girls FT3 showed a significant negative association with PFOS(branched) [1].

Natural hormones and receptor-binding endocrine disrupting chemicals tend to display, in arithmetical plots, in relation with primary effects such as changes in gene expression, an asymptotic-like dose-response curve with an almost linear steep response at very low doses, followed by saturation at higher concentrations reflected in the fact that the dose response curve becomes almost horizontal [2]. Given the likelihood that the endocrine disrupting effects that we observed were due to binding of PFAS to receptors (see discussion), we set out, in this follow-up study, to test a work hypothesis stating that the strength of the observed associations would be stronger below the median PFAS serum concentrations than above the median PFAS serum concentrations. We tested this for 84 (those calculated for continuous PFAS concentrations) of the 85 significant associations between PFAS and health effects that were described in our publication by van Larebeke et al. [1], publication that is referred to in our present publication as the “original study”. Exposure-effect curves were also investigated using distance weighted least square graphs and for 5 of the most relevant PFAS parameters also through continuous piecewise linear models and multiple regressions including quadratic PFAS concentration terms.

## Main text

### Study population

As described in detail by van Larebeke et al. [1] the study population included 303 adolescents (12.55 to 16.64 years of age, with 60% aged between 13.5 and 15.5) residing in a circular area with a radius of 5 km round the center of the 3 M site in Zwijndrecht, Belgium. Sufficient data on exposure, outcome and important covariates could be collected for 146 female and 139 male adolescents.

### Collection of samples and questionnaires

As described in detail by van Larebeke et al. [1], collection of samples and measurement of height and weight of participants occurred between 28 June and 31 August 2022. All male adolescents were sampled before 11 a.m. to account for the diurnal variation in sex hormones. The blood samples were immediately processed and aliquoted and stored at − 80 °C within 12 h for PFAS analysis or analyzed for hormones within 24 h after sampling. Participants filled out digital questionnaires on socio-demographic, life style and environmental characteristics.

### Measurements of PFAS in blood

As described in detail by van Larebeke et al. [1] PFAS compounds were measured in serum according to the Flemish reference method (WAC/IV/A/025; https://emis.vito.be/nl/erkende-laboratoria/water-gop/compendium-wac).The method quality is ensured through a Belgian accreditation system, the (BELAC) ISO 17,025 accreditation.

The following PFAS parameters were considered in this Comment: linear Perfluorobutanoic acid (PFBA), linear Perfluorooctanoic acid (PFOA), linear + branched Perfluorooctanoic acid (PFOAtotal), linear Perfluorononanoic acid (PFNA), linear Perfluorodecanoic Acid (PFDA), linear Perfluorohexanesulfonic acid (PFHxS), linear + branched Perfluorohexanesulfonic acid (PFHxStotal), linear Perfluorooctanesulfonic acid (PFOS), linear + branched Perfluorooctanesulfonic acid (PFOStotal) and branched Perfluorooctanesulfonic acid (PFOSbranched) calculated by subtracting linear PFOS from PFOS (total).

In addition, 3 sum parameters were calculated as accounted for in the “original study”: the sum of the linear PFOA, PFNA, PFHxs, and PFOS (referred to as 4PFAS), the sum of total PFOA, linear PFNA, total PFHxS, and total PFOS (referred to as lb4PFAS), and the sum of linear N-Methylperfluorooctanesulfonamide (Me PFOSAA), linear PFHxS, total PFOA, linear PFDA, linear Perfluoroundecanoic acid (PFUnDA), total PFOS, and linear PFNA (referred to as 7PFAS).

### Measurement of thyroid and sex hormones

Sex and thyroid hormones were measured by an accredited clinical lab as described by van Larebeke et al. [1]. Thyroid-stimulating hormone mIU/L (TSH) and free triiodothyronine ng/dL (FT3), were measured in females and males. Sex hormones were measured in males only: testosterone (ng/dL) (TT), bioavailable testosterone (ng/dL) (bioavailable TT), sex hormone-binding globulin (nmol/L) (SHBG), luteinizing hormone (IU/L) (LH), progesterone (µg/L) (P4). Hormone concentrations were > LOD/LOQ for all samples and markers, except for P4, which was detected in only 48% of the samples, and was further analyzed as a binary variable (values > = LOD versus values < LOD).

### Assessment of pubertal development parameters

Assessment of pubertal development parameters was described in detail by Van Larebeke et al. [1]. Height of the participants was measured in cm by a study nurse during the field work. Puberty development as described by Marshall and Tanner was estimated using a standardized questionnaire (Pubertal Development Scale) in which the participant self-assessed its stage of development for: growth spurt; skin changes, as at puberty the skin of children acquires the characteristics of adult skin; body hair (excluding head); voice changes (for males); facial hair (for males); breast development (for females); age of first period (menarche). For these variables 3 categories were used in statistical models.

Pubertal development scale (PDS) was assessed in a sex specific manner. Here we report only results pertaining to age- standardized puberty development for female adolescents. Age standardized PDS was calculated as a binary variable indicating whether the expected stage for age was reached or not. To be categorized as having reached the expected stage for age, females < 11.85 years, [11.85– 12.75] years, [12.75–14.65] years, and > 14.65 years should have reached early puberty, mid-puberty, late puberty, and post puberty, respectively.

### Statistical analysis

Exposure-effect curves for all associations reported as significant by Van Larebeke et al. [1] were visually inspected using Distance Weighted Least Square Graphs produced using Statistica 14.0. This method provides a non‑parametric smoothed curve by weighting observations according to their distance from each estimation point.

The present study is an extended analysis of 84 statistically significant associations between exposure and outcome assessed by Van Larebeke et al. [1]. For each exposure parameter, the study population was split in a lower exposed subpopulation (below the median) and a higher exposed subpopulation. The median concentration (P50) of each exposure parameter is presented in Table 1. The strength of the association was compared between both subpopulations based on the regression coefficients.

**Table 1:** PFAS Serum concentrations in µg/L for the total adolescent population included in the study

PFAS	*n*	%>=LOQ	*P*^c^10	*P*^c^25	*P*^c^50	*P*^c^75	*P*^c^ 90
4PFAS^a^	283^a^	100^a^	2.35	3.17	4.40	7.80	20.77
lb4PFAS^a^	283^a^	100^a^	4.97	6.60	9.44	14.98	30.33
7PFAS^a b^	283^a^	100^a, b^	5.19	6.80	9.65	15.33	30.60
PFHxS	283	100	0.29	0.36	0.51	0.84	1.50
PFHxS(total)	283	100	0.31	0.38	0.54	0.88	1.50
PFOA	283	100	0.70	0.87	1.10	1.40	1.80
PFOA(total)	283	100	0.73	0.92	1.20	1.50	1.90
PFBA	283	72	0.065	0.10	0.15	0.19	0.35
PFDA	283	73	0.066	0.10	0.14	0.200	0.28
PFOS	283	100	0.97	1.400	2.40	5.20	18.00
PFOS(total)	283	100	3.20	4.70	7.10	13.00	28.00
PFOS(branched)	283	100	1.80	2.60	4.20	6.90	10.00
PFNA	283	99	0.15	0.19	0.26	0.34	0.45

^a^Sum parameters were calculated for all participants. For the sum parameters data concerning PFNA were lacking for 2 participants

^b^For 7PFAS, the values below LOQ in the individual parameters were imputed by the LOQ/sqrt(2), since the quentification rate of MePFOSAA and PFUnDA was below 30%

^c^Percentile

To allow for a comparison between associations of biological/health effects with PFAS concentrations on the one hand below median serum concentrations and on the other above median serum concentrations, original PFAS concentrations without log transformation were used in linear multiple regression with continuous effect parameters without log transformation, logistic models with binary effect parameters, and Ordinal Multinomial Probit models with ordinal effect parameters. As in the study by van Larebeke et al. [1], all multiple regressions performed in this follow-up study were performed for males and females separately, and age and “Making ends meet with the income – manage to live comfortably” were included as covariates in all models. The analysis of residuals was satisfactory. Regression coefficients, their 95% confidence limits, the standard errors, p values, the width of the 95% confidence intervals and the correlations between different PFAS parameters limited to concentrations below median values or after exclusion of concentrations below median values (designated respectively as “below the median” and “above the median”) were calculated with the Statistica 14.0 program. Significance of the difference between an regression coefficient observed for an association below the median and an regression coefficient observed for an association above the median were assessed using a two- tailed Wald test. The percentiles 10, 25, 50, 75 and 90 as measured for all of the participating adolescents, of PFAS compounds involved in the present study are shown in Table 1. Histograms of continuous effect parameters for the total participating adolescent population are shown in supplementary material Figures S1, S2, S3, S4, S5, S6, S7 and S8.

Using Statistica 14.0, exposure-effect relationships for 5 of the most relevant PFAS parameters (PFOA, PFHxS, PFOS, 4PFAS and lb4PFAS) were also investigated through multiple regressions including quadratic PFAS concentration terms and through continuous piecewise linear models for male and female adolescents separately. In both these type of models age and “Making ends meet with the income – manage to live comfortably” were included as covariates. Based on the PFAS serum concentration percentiles, the concentration ranges studied for all five PFAS parameters were “< p10”, ”p10 to< p25”, “p25 to< p50”, “p50 to< p75”, “p75 to < p 90” and “p90 and higher”.

## Results

Although the observed median PFAS serum concentrations in the studied cohort were quite low in comparison with the present health based guidance values, the regression coefficient for the restricted association below the median PFAS serum concentration was stronger than the regression coefficient for the corresponding restricted association above the median in 72 of the 84 associations as can be seen from Table 2. As several PFAS variables exhibit highly skewed distributions with a small number of extremely high values which might have a disproportionate influence on the slope above the median serum concentration a sensitivity analysis was performed on the 9 associations showing a stronger slope below the median and involving PFOS or 4PFAS, the PFAS parameters for which the most extreme values were found. After exclusion of values equal or superior to the 97 percentile all associations remained weaker above the median and for 8 of these associations the exclusion of the highest PFAS values further weakened the slope slightly, only for one association the slope became slightly stronger but still much weaker than below the median (see supplementary materials Table S1 for details). As shown in the last column of Table 2, the regression coefficient below the median is often much stronger than the one above the median, for 38 associations at least ten times stronger.

**Table 2:** Comparison between the regression coefficients below and above the median PFAS serum concentrations

Effect^a^	PFAS µg/L^b^	Sign^c^	Below median^d^					Above median ^e^							
			Regression coefficient (odds ratio if relevant)	Standard error	Regression coefficient lower 95% CI (odds ratio if relevant)	Regression coefficient upper 95%CI (odds ratio if relevant)	*p*	Regression coefficient	Standard error	Regression coefficient lower 95% CI (odds ratio if relevant)	Regression coefficient upper 95% CI (odds ratio if relevant)	*p*	*n*	*P* difference^f^	Ratio Regression coefficient below/ Regression coefficient above
Observations on male adolescents															
Height cm	PFOA	Neg.	0.14	4.72	-9.11	9.39	0.98	-0.37	1.17	-2.66	1.93	0.75	*147*	*0.92*	-0.38^g^
FT3 ng/dL	PFOA	Pos	0.0329	0.0167	0.0002	0.0656	0.048	0.0100	0.0050	0.0003	0.0197	0.043	*147*	*0.19*	3.29
Total Testosterone ng/dL	PFOA	Neg.	-123.1	140.2	-397.8	151.7	0.39	-13.5	30.0	-72.2	45.2	0.65	*146*	*0.45*	9.12
Bioavailable testosteron ng/dL	PFOA	Neg.	-87.8	52.7	-191.0	15.5	0.096	-9.6	13.7	-36.4	17.3	0.49	*146*	*0.15*	9.15
LH IU/L	PFOA	Neg.	-0.47	0.94	-2.32	1.38	0.62	-0.08	0.16	-0.40	0.24	0.61	*146*	*0.68*	5.88
Latent OMP score^i^ for Growth Spurt	PFOA	Neg.	-0.39	0.93	-2.20	1.43	0.68	-0.14	0.24	-0.61	0.33	*0.57*	*136*	*0.79*	2.79
Height cm	PFOA(total)	Neg.	-1.64	4.03	-9.55	6.26	0.68	-0.09	1.10	-2.25	2.07	0.94	*147*	*0.71*	18.22
FT3 ng/dL	PFOA(total)	Pos	0.0418	0.0146	0.0132	0.0705	0.0042	0.0113	0.0045	0.0024	0.0201	0.012	*147*	*0.20*	3.70
Totaal Testosteron ng/dL	PFOA(total)	Neg.	-74.7	148.3	-365.3	216.0	0.61	-11.3	26.5	-63.2	40.6	0.67	*146*	*0.67*	6.61
Bioavailable testosteron ng/dL	PFOA(total)	Neg.	-86.7	55.6	-195.7	22.3	0.12	-8.1	12.1	-31.9	15.6	0.50	*146*	*0.17*	10.70
LH IU/L	PFOA(total)	Neg.	-0.55	0.99	-2.48	1.38	0.57	-0.06	0.14	-0.34	0.22	0.68	*146*	*0.62*	9.17
Latent OMP score^i^ for Growth Spurt	PFOA(total)	Neg.	-0.05	0.94	-1.90	1.80	0.96	-0.12	0.22	-0.55	0.30	*0.57*	*136*	*0.93*	0.42
FT3 ng/dL	PFHxS	Pos	0.109	0.040	0.030	0.187	0.0069	0.0063	0.0059	-0.0052	0.0178	0.28	*147*	*0.012*	17.30
Totaal Testosteron ng/dL	PFHxS	Neg.	-522.3	268.3	-1048.2	3.5	0.052	-16.8	34.7	-84.9	51.3	0.63	*146*	*0.063*	31.09
Bioavailable testosteron ng/dL	PFHxS	Neg.	-347.6	105.0	-553.4	-141.8	0.00093	-12.9	15.8	-43.8	18.1	0.41	*146*	*0.0018*	26.95
SHBG nmol/L	PFHxS	Pos.	18.0	22.1	-25.3	61.3	0.42	4.09	3.84	-3.43	11.62	*0.29*	*146*	*0.43*	4.40
LH IU/L	PFHxS	Neg.	-1.47	1.81	-5.03	2.08	0.42	0.0008	0.1738	-0.3400	0.3420	1.00	*146*	*0.42*	-1837^g^
Latent OMP score^i^ for Growth Spurt	PFHxS	Neg.	-2.00	1.86	-5.65	1.64	0.28	-0.03	0.26	-0.54	0.49	0.92	*136*	*0.29*	66.67
Latent OMP score^j^ for Body Hair growth	PFHxS	Neg.	-4.30	1.93	-8.08	-0.52	0.026	-0.250	0.254	-0.747	0.247	0.32	*146*	*0.038*	17.20
FT3 ng/dL	PFHxS(total)	Pos	0.132	0.041	0.051	0.213	0.0014	0.0067	0.0056	-0.0043	0.0176	0.23	*147*	*0.0029*	19.70
Totaal Testosteron ng/dL	PFOA(total)	Neg.	-74.7	148.3	-365.3	216.0	0.61	-11.3	26.5	-63.2	40.6	0.67	*146*	*0.67*	6.61
Bioavailable testosteron ng/dL	PFHxS(total)	Neg.	-305.3	112.8	-526.4	-84.2	0.0068	-20.6	20.4	-60.6	19.5	0.31	*146*	*0.014*	14.82
LH IU/L	PFHxS(total)	Neg.	-1.41	1.87	-5.06	2.25	0.45	-0.034	0.173	-0.373	0.305	0.84	*146*	*0.46*	41.47
Latent OMP score^i^ for Growth Spurt	PFHxS(total)	Neg.	-1.10	1.92	-4.86	2.65	0.57	-0.01	0.25	-0.51	0.49	*0.96*	*136*	*0.57*	110.00
Latent OMP score^j^ for Body Hair growth	PFHxS(total)	Neg.	-4.48	1.86	-8.13	-0.83	0.016	-0.257	0.249	-0.744	0.231	0.30	*146*	*0.026*	17.43
Bioavailable testosteron ng/dL	PFOS	Neg.	7.71	23.68	-38.70	54.11	0.74	-0.58	0.51	-1.57	0.42	0.26	*146*	*0.73*	-13.29^g^
SHBG nmol/L	PFOS	Pos.	2.37	4.08	-5.62	10.36	0.56	0.10	0.14	-0.18	0.38	0.49	*146*	*0.63*	23.70
LH IU/L	PFOS	Neg.	-0.515	0.330	-1.162	0.132	0.12	-0.009	0.006	-0.021	0.003	0.16	*146*	*0.13*	57.22
Latent score^h^ in binary logit model for Progestrone above limit of detection	PFOS	Neg.	-0.042 (0.959)	0.379	-0.785 (0.456)	0.702 (2.018)	0.91	-0.0067 (0.993)	0.0073	-0.0209 (0.979)	0.0076 (1.008)	*0.36*	*146*	*0.93*	6.27
Latent OMP score^i^ for Growth Spurt	PFOS	Neg.	0.010	0.327	-0.630	0.650	0.98	-0.007	0.010	-0.026	0.011	0.45	*136*	*0.95*	-1.43^g^
Latent OMP score^j^ for Body Hair growth	PFOS	Neg.	-0.408	0.339	-1.072	0.256	0.23	-0.005	0.010	-0.024	0.014	0.60	*146*	*0.24*	81.60
Height cm	PFOS(branched)	Neg.	-1.40	0.94	-3.24	0.44	0.14	-0.28	0.21	-0.68	0.13	0.18	*147*	*0.24*	5.00
LH IU/L	PFOS(branched)	Neg.	0.021	0.171	-0.314	0.356	0.90	-0.034	0.026	-0.085	0.018	0.20	*146*	*0.75*	-0.62^g^
Height cm	PFOS(total)	Neg.	-1.070	0.627	-2.298	0.159	0.087	-0.045	0.046	-0.135	0.044	0.32	*147*	*0.10*	23.78
LH IU/L	PFOS(total)	Neg.	-0.082	0.108	-0.294	0.129	0.45	-0.009	0.006	-0.020	0.002	0.12	*146*	*0.50*	9.11
Latent score^h^ in binary logit model for Progestrone above limit of detection	PFOS(total)	Neg.	0.030 (1.030)	0.121	-0.206 (0.814)	0.267 (1.306)	0.80	-0.0062 (0.994)	0.0066	-0.0191 (0.981)	0.0067 (1.007)	*0.35*	*146*	*0.76*	-4.84^g^
Latent OMP score^i^ for Growth Spurt	PFOS(total)	Neg.	-0.103	0.106	-0.311	0.106	0.33	-0.005	0.009	-0.022	0.012	0.55	*136*	*0.36*	20.60
Latent OMP score^j^ for Body Hair growth	PFOS(total)	Neg.	-0.047	0.105	-0.253	0.159	0.66	-0.007	0.009	-0.024	0.010	0.42	*146*	*0.71*	6.71
TSH mIU/L	PFDA	Neg.	-4.17	3.96	-11.92	3.59	0.29	-0.73	0.73	-2.15	0.70	0.32	*147*	*0.39*	5.71
SHBG nmol/L	PFDA	Pos.	122.8	68.9	-12.3	257.9	0.075	14.8	14.1	-12.7	42.4	0.29	*146*	*0.13*	8.30
LH IU/L	PFDA	Neg.	-6.17	5.20	-16.36	4.01	0.23	-1.31	2.70	-6.60	3.99	0.63	*146*	*0.41*	4.71
Latent OMP score^i^ for Growth Spurt	PFDA	Neg.	-2.22	5.45	-12.90	8.47	0.68	-2.98	1.22	-5.37	-0.60	*0.014*	*136*	*0.89*	0.74
FT3 ng/dL	PFNA	Pos	0.137	0.081	-0.022	0.295	0.092	0.022	0.029	-0.034	0.078	0.45	*147*	*0.18*	6.23
SHBG nmol/L	PFNA	Pos.	114.4	44.9	26.4	202.4	0.011	41.7	17.0	8.5	75.0	0.014	*146*	*0.13*	2.74
LH IU/L	PFNA	Neg.	-4.59	3.89	-12.22	3.04	0.24	-0.97	0.89	-2.72	0.78	0.28	*146*	*0.37*	4.73
Latent OMP score^i^ for Growth Spurt	PFNA	Neg.	-3.59	4.00	-11.44	4.26	0.37	-2.81	1.20	-5.16	-0.46	*0.019*	*136*	*0.85*	1.28
Latent OMP score^j^ for Body Hair growth	PFNA	Neg.	-3.09	3.93	-10.78	4.61	0.43	-2.72	1.21	-5.10	-0.34	0.025	*146*	*0.93*	1.14
Latent score^h^ in binary logit model for Progestrone above limit of detection	PFBA	Pos.	11.10 (66. 10^3^)^o^	5.54	0.25 (1.284)	21.95 (34.10^8^)^o^	0.045	-1.07 (0.343)	1.25	-3.51 (0.030)	1.37 (3.935)	*0.39*	*146*	*0.033*	-10.37^g^
Bioavailable testosteron ng/dL	4PFAS	Neg.	-5.07	14.43	-33.36	23.22	0.73	-0.54	0.49	-1.51	0.43	0.28	*146*	*0.75*	9.39
SHBG nmol/L	4PFAS	Pos.	4.36	2.56	-0.65	9.38	0.088	0.14	0.13	-0.12	0.39	0.30	*146*	*0.10*	31.14
LH IU/L	4PFAS	Neg.	-0.283	0.205	-0.685	0.118	0.17	-0.007	0.006	-0.019	0.004	0.22	*146*	*0.18*	40.43
Latent score^h^ in binary logit model for Progestrone above limit of detection	4PFAS	Neg.	0.059 (1.061)	0.214	-0.360 (0.698)	0.478 (1.613)	0.78	-0.0092 (0.991)	0.0074	-0.0236 (0.977)	0.0053 (1.005)	*0.21*	*146*	*0.75.*	-6.41^g^
Latent OMP score^i^ for Growth Spurt	4PFAS	Neg.	-0.193	0.208	-0.600	0.215	0.35	-0.004	0.009	-0.021	0.014	0.69	*136*	*0.36*	48.25
Latent OMP score^j^ for Body Hair growth	4PFAS	Neg.	-0.403	0.211	-0.817	0.010	0.056	-0.0072	0.0090	-0.0249	0.0105	0.40	*146*	*0.062*	55.97
Height cm	Lb4PFAS	Neg.	-1.207	0.611	-2.404	-0.011	0.047	-0.048	0.042	-0.130	0.035	0.26	*147*	*0.059*	25.15
Bioavailable testosteron ng/dL	Lb4PFAS	Neg.	-12.62	7.24	-26.80	1.57	0.081	-0.590	0.435	-1.442	0.261	0.17	*146*	*0.098*	21.39
LH IU/L	Lb4PAS	Neg.	-0.150	0.105	-0.355	0.055	0.15	-0.0103	0.0054	-0.0208	0.0002	0.055	*146*	*0.18*	14.56
Latent score^h^ in binary logit model for Progestrone above limit of detection	Lb4PAS	Neg.	0.017 (1.017)	0.103	-0.185 (0.831)	0.219 (1.245)	0.87	-0.0082 (0.992)	0.0067	-0.0213 (0.979)	0.0049 (1.005)	*0.22*	*146*	*0.81*	-2.07^g^
Latent OMP score^i^ for Growth Spurt	Lb4PFAS	Neg.	-0.189	0.103	-0.391	0.012	0.065	-0.0068	0.0081	-0.0227	0.0091	0.40	*136*	*0.058*	27.79
Latent OMP score^j^ for Body Hair growth	Lb4PFAS	Neg.	-0.125	0.101	-0.323	0.074	0.22	-0.009	0.008	-0.025	0.008	0.30	*146*	*0.25*	13.89
Height cm	7PFAS	Neg.	-1.188	0.604	-2.372	-0.004	0.049	-0.048	0.042	-0.130	0.035	0.26	*147*	*0.061*	24.75
Bioavailable testosteron ng/dL	7PFAS	Neg.	-11.98	7.17	-26.04	2.08	0.094	-0.59	0.43	-1.44	0.26	0.18	*146*	*0.11*	20.31
LH IU/L	7PFAS	Neg.	-0.146	0.103	-0.349	0.057	0.16	-0.0103	0.0053	-0.0208	0.0002	0.054	*146*	*0.19*	14.17
Latent score^h^ in binary logit model for Progestrone above limit of detection	7PFAS	Neg.	-0.003 (0.997)	0.100	-0.200 (0.819)	0.194 (1.214)	0.98	-0.0085 (0.992)	0.0067	-0.0216 (0.979)	0.0046 (1.005)	*0.21*	*146*	*0.99*	0.35
Latent OMP score^i^ for Growth Spurt	7PFAS	Neg.	-0.187	0.102	-0.386	0.013	0.066	-0.0069	0.0081	-0.0228	0.0089	0.39	*136*	*0.059*	27.10
Latent OMP score^j^ for Body Hair growth	7PFAS	Neg.	-0.125	0.100	-0.321	0.071	0.21	-0.009	0.008	-0.025	0.008	0.30	*146*	*0.25*	13.89
Observations on female adolescents															
Latent score^k^ in binary logit model for reaching Pubertal Stage expected for age	PFOA	Neg.	-3.38 (0.034)	1.79	-6.89 (0.001)	0.13 (1.139)	0.059	-1.41 (0.244)	0.94	-3.25 (0.039)	0.44 (1.553)	*0.14*	*139*	*0.33*	2.40
Latent OMP score^i^ for Growth Spurt	PFOA	Neg.	-0.96	1.03	-2.97	1.06	0.35	-0.33	0.49	-1.30	0.63	*0.50*	*133*	*0.59*	2.91
Latent OMP score^j^ for Body Hair growth	PFOA	Neg.	-1.19	0.90	-2.94	0.57	0.19	-0.42	0.45	-1.31	0.47	0.35	*146*	*0.45*	2.83
Latent OMP score^m^ for Breast Development	PFOA	Neg.	-1.96	0.96	-3.85	-0.07	0.043	0.52	0.48	-0.43	1.47	0.28	*142*	*0.022*	-3.77^g^
Latent score^k^ in binary logit model for reaching Pubertal Stage expected for age	PFOA(total)	Neg.	-1.98 (0.138*)*	1.79	-5.48 (0.004)	1.53 (4.618)	0.27	-0.92 (0.399*)*	0.83	-2.56 (0.077)	0.71 (2.034)	*0.27*	*139*	*0.59*	2.15
Latent OMP score^i^ for Growth Spurt	PFOA(total)	Neg.	-0.27	0.85	-1.94	1.40	0.75	0.02	0.54	-1.05	1.08	*0.97*	*133*	*0.78*	-13.50^g^
Latent OMP score^j^ for Body Hair growth	PFOA(total)	Neg.	-0.27	0.94	-2.11	1.58	0.78	-0.09	0.42	-0.90	0.73	0.83	*146*	*0.86*	3.00
Latent OMP score^m^ for Breast Development	PFOA(total)	Neg.	-1.61	0.99	-3.55	0.34	0.11	0.44	0.45	-0.45	1.32	0.34	*142*	*0.062*	-3.66^g^
Latent score^k^ in binary logit model for reaching Pubertal Stage expected for age	PFHxS	Neg.	-5.40 (0.005)	3.11	-11.49 (0.000)	0.69 (1.994)	0.082	-0.075 (0.928)	0.244	-0.552 (0.576)	0.403 (1.496)	*0.76*	*139*	*0.089*	72.00
Latent OMP score^j^ for Body Hair growth	PFHxS	Neg.	-1.65	1.57	-4.73	1.43	0.29	-0.07	0.12	-0.31	0.16	0.54	*146*	*0.32*	23.57
Latent score^k^ in binary logit model for reaching Pubertal Stage expected for age	PFHxS(total)	Neg.	-5.06 (0.006*)*	2.92	-10.78 (0.000)	0.67 (1.954)	0 0.083	-0.070 (0.932)	0.236	-0.533 (0.587)	0.392 (1.480)	*0.77*	*139*	*0.090*	72.29
Latent OMP score^j^ for Body Hair growth	PFHxS(total)	Neg.	-1.38	1.44	-4.21	1.45	0.34	-0.08	0.12	-0.31	0.15	0.49	*146*	*0.37*	17.25
Latent score^k^ in binary logit model for reaching Pubertal Stage expected for age	PFOS	Neg.	0.527 (1.694)	0.589	-0.627 (0.534)	1.682 (5.376)	0.37	-0.007 (0.993)	0.010	-0.027 (0.973)	0.014 (1.014)	*0.52*	*139*	*0.37*	-75.29^g^
FT3 ng/dL	PFOS(branched)	Neg.	0.109	0.040	0.030	0.187	0.0069	-0.0004	0.0019	-0.0041	0.0032	*0.81*	*147*	*0.0071*	-272.5^g^
Latent OMP score^i^ for Growth Spurt	PFOS (branched	Pos.	0.320	0.171	-0.020	0.655	0.061	0.024	0.042	-0.060	0.107	0.56	*133*	*0.095*	13.33
Latent OMP score^l^ for Pubertal Skin changes	PFOS(branched)	Pos.	0.343	0.174	0.003	0.684	0.048	-0.009	0.038	-0.084	0.067	0.82	*144*	*0.049*	-38.11^g^
Latent OMP score^l^ for Pubertal Skin changes	PFOS(total)	Pos.	0.138	0.097	-0.052	0.328	0.158	0.002	0.006	-0.011	0.014	0.80	*144*	*0.16*	69.00
Latent score^k^ in binary logit model for reaching Pubertal Stage expected for age	4PFAS	Neg.	-0.053	0.319	-0.679	0.572	0.87	-0.004	0.009	-0.022	0.015	*0.69*	*139*	*0.88*	13.25

^a^Effect parameter as used in van Larebeke et al.(2025)

^b^Parameter of internal exposure as used in van Larebeke et al.(2025)

^c^Sign of the statistically significant association as described in van Larebeke et al.(2025). Negative if higher PFAS serum concentrations are associated with lower values of the effect parameter

^d^Data describing the association limited to PFAS serum values below the median value

^e^Data describing the association after exclusion of PFAS serum values below the median value

^f^p value for the difference between the regression coefficients below the median and above the median PFAS serum concentration

^g^Ratio Regression coefficient below/ Regression coefficient above is negative if the regression coefficients below and above the median have a different sign implying that their strength cannot be directly compared

^h^The change in the latent score in a Binomial logit model per increase of 1 µg/L PFAS is the regression coefficient for the effect of the PFAS concentration on reaching a level of progesterone above the limit of detection

^i^The change in the latent score in an Ordinal Multinomial Probit model per increase of 1 µg/L PFAS is the regression coefficient for the effect of the PFAS concentration on reaching a further stage of growth spurt

^j^The change in the latent score in an Ordinal Multinomial Probit model per increase of 1 µg/L PFAS is the regression coefficient for the effect of the PFAS concentration on reaching a further stage of body hair growth

^k^The change in the latent score in a Binomial logit model per increase of 1 µg/L PFAS is the regression coefficient for the effect of the PFAS concentration on reaching the Pubertal stage expected for age

^l^The change in the latent score in an Ordinal Multinomial Probit model per increase of 1 µg/L PFAS is the regression coefficient for the effect of the PFAS concentration on reaching a further stage of pubertal skin change

^m^The change in the latent score in an Ordinal Multinomial Probit model per increase of 1 µg/L PFAS is the regression coefficient for the effect of the PFAS concentration on reaching a further stage of breast development

^n^Change in the odds ratio for reaching the developmental stage in question (that is reaching a level of progesterone above the limit of detection or reaching the Pubertal stage expected for age)

^o^These extreme values calculated for 1 µg/L are linked to the fact that a significant positive association was observed by van Larebeke et al.(2025) for an interquartile PFBA concentration of 0.09 µg/L

In boys, the positive associations between PFHxS and PFHxS(total) with FT3 were significantly stronger for the lower exposed subpopulation (below median), while in the same subpopulation a rise in PFHxS or (PFHxS(total) was associated with a significantly steeper drop in biological available testosterone and a significantly steeper drop of body hair growth compared to the subpopulation that was exposed at levels above the median. The negative associations of PFHxS and PFHxS(total) with reaching pubertal stage expected for age were marginally stronger below median serum concentration.

The negative associations of PFOA and PFOA(total) with breast development in girls were respectively significantly and marginally significantly stronger for concentrations below the median than above.

The negative associations of lb4PFAS and 7 PFAS with growth spurt and height for boys were marginally stronger below median PFAS concentrations than above.

The negative associations of 4PFAS with body hair development in boys and of lb4PFAS with bioavailable testosterone in boys were marginally stronger below median PFAS concentrations than above.

The positive associations of PFOS(branched) with pubertal skin changes, growth spurt and FT3 in girls were respectively significantly, marginally significantly and significantly stronger below the median than above.

The positive association of PFBA with reaching progesterone serum levels above LOQ in boys was significantly stronger below the median than above.

**Fig. 1:**
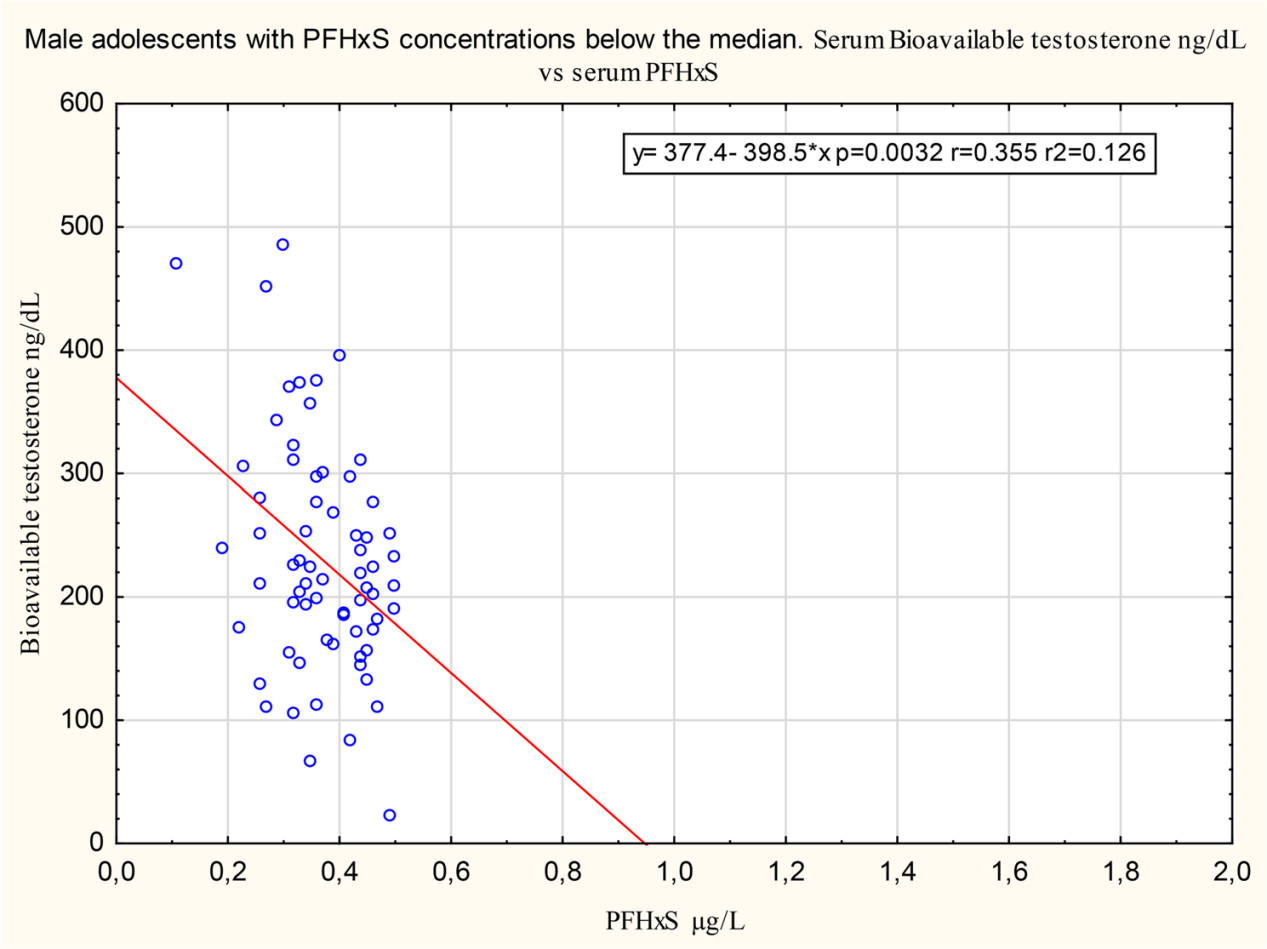
Association of serum concentrations of PFHxS and bioavailable testosterone below the median. The association between serum concentrations of PFHxS and bioavailable testosterone below the median PFHxS concentration, on a scale that permits direct comparison with the corresponding association above the median PFHxS concentration

**Fig. 2:**
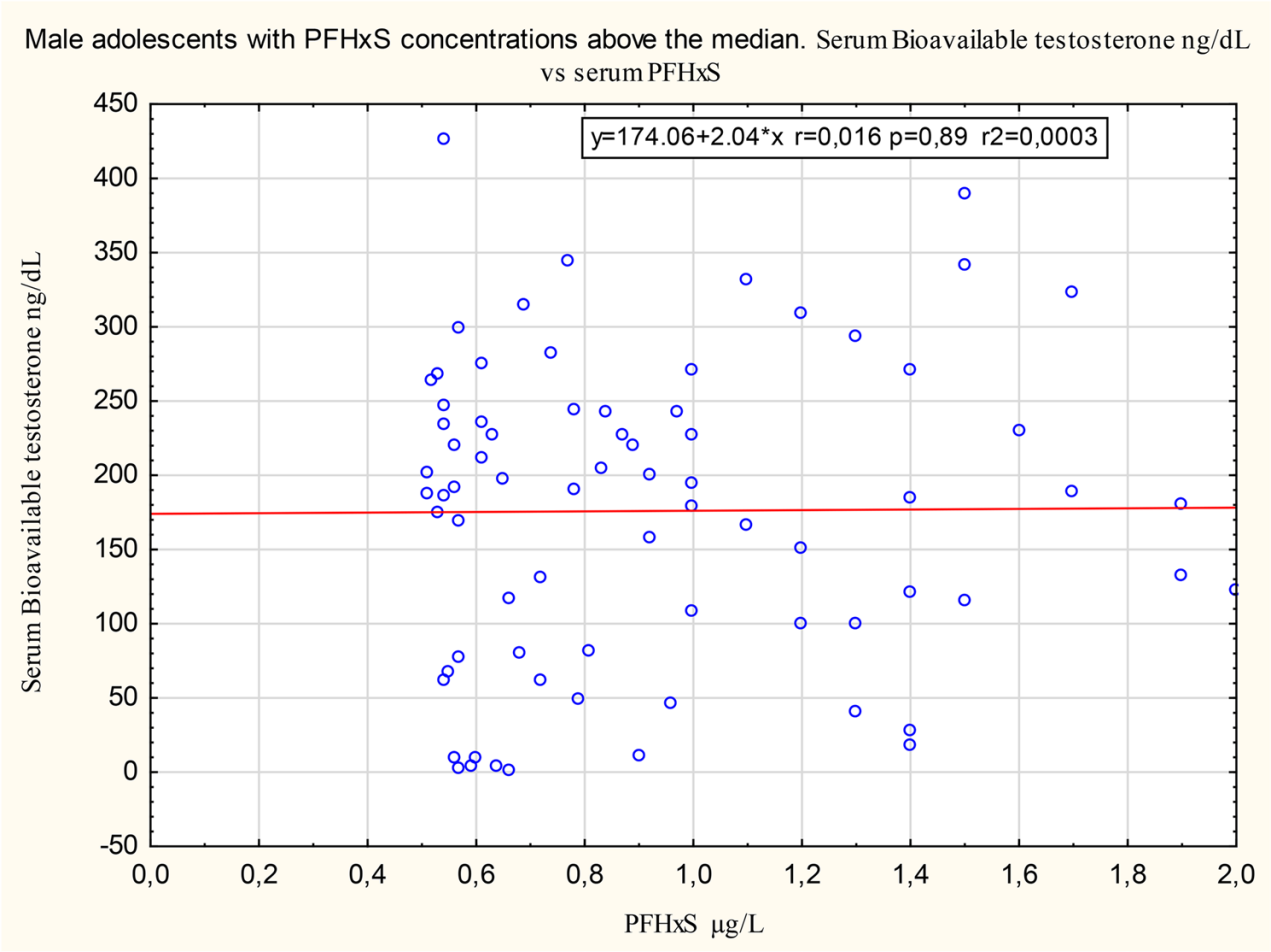
Association of serum concentrations of PFHxS and bioavailable testosterone above the median. The association between serum concentrations of PFHxS and bioavailable testosterone above the median PFHxS concentration, on a scale that permits direct comparison with the corresponding association below the median PFHxS concentration

Figures1 and 2 show, for illustration purposes interesting, comparisons between associations below and above the median for the association of PFHxS with bioavailable testosterone. Supplementary material Figures S9 to S12 show comparisons between associations below and above the median for the association of PFOA and breast development.

As shown in Table 2, the regression coefficients in restricted associations below the median are significantly stronger than those in restricted associations above the median for 9 associations, and marginally significantly stronger for 11 associations. In only 1 restricted association above the median the regression coefficient is significantly stronger than that in the restricted association below the median and no marginally significant stronger restricted associations above the median are observed.

As shown in Table 2, in 15 associations the regression coefficients of the restricted association below the median and the regression coefficient of the restricted association above the median had a different sign. In 9 of these associations the regression coefficient above the median was stronger.

As shown in Table 2, PFAS concentrations below the median concentrations showed 14 significant and 16 marginally significant associations with biological/health effect parameters. As shown in Table 2 PFAS concentrations above the median concentrations showed 6 significant and 2 marginally significant associations with biological/health effect parameters.

From Table 2 one also gets the impression that the regression coefficients of restricted associations below the median PFAS concentration show often much higher standard errors and 95% confidence limit widths. Table 3 shows that this is indeed the case. In all of the 84 associations the standard errors and the width of the 95% confidence intervals of the regression coefficients were larger below the median PFAS concentrations than above the median PFAS concentrations, suggesting that PFAS measurements were less precise below the median serum concentration.

**Table 3:** Regression coefficients. Ratio’s of standard errors and width of confidence intervals below/above the median^a^

	*n*	Mean	Median	Minimum	Maximum	Lower quartile	Upper quartile	Percentile 10	Percentile 90
Ratio standard error below/ standard error above	84	12.75	7.569	1.570	56.81	4.361	15.99	2.264	29.19
Ratio width 95%CI below/ width 95%CI above	84	12.02	7.513	1.570	56.82	4.361	15.07	2.193	29.00

^a^Descriptive statistics, for all of the 84 studied associations, of the ratio’s between respectively the standard error of the regression coefficient and the width of the 95% confidence interval in the restricted association below the median and the standard error of the regression coefficient and the width of the 95% confidence interval in the corresponding restricted association above the median

**Fig. 3:**
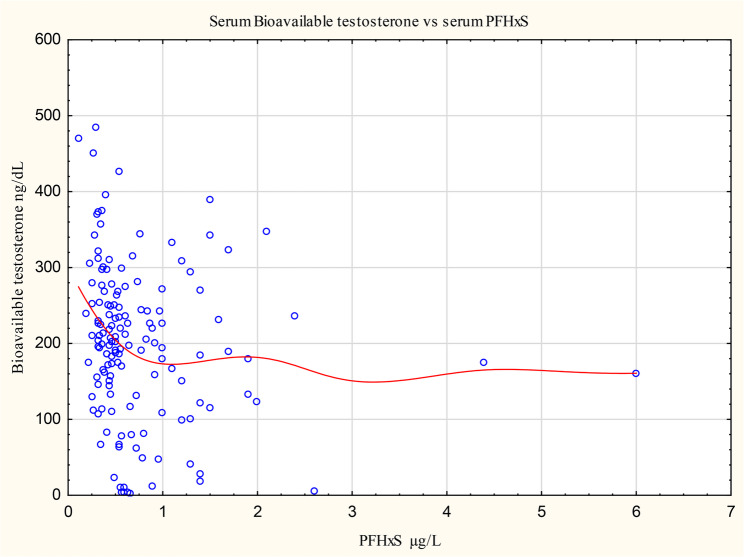
Distance Weighted Least Square Graph of the Association of serum concentrations of PFHxS and bioavailable testosterone. At low PFHxS concentrations the Distance Weighted Least Square curve shows a pronounced downward slope, which disappears at higher concentrations

The Distance Weighted Least Square Graphs, shown in the supplementary materials Figures S13, for all associations concerning male adolescents, and Figures S14, for all associations concerning female adolescents, reported as significant by van Larebeke et al.(2025) [1], support the notion that the slope of the curve consistent with the significant effect reported by van Larebeke et al.(2025) [1] is often stronger at low PFAS serum concentrations than at higher concentrations. Figure 3 shows for illustration an interesting example concerning the association between PFHxS serum concentration and bioavailable testosterone. However, the Distance Weighted Least Square curves do not provide meaningful information at their right end due to the limited number of data points on which they rest there.

The results of multiple regressions entailing linear and quadratic PFAS concentration terms for 5 of the most relevant PFAS parameters are shown in supplementary materials Table S2.The quadratic term was found to be significant in six and marginally significant in two of the 39 studied regressions entailing both a linear and quadratic term for PFAS concentration, indicating in those associations the existence of non-linearity. In 35 of the 39 studied regressions the quadratic term had a sign opposed to the sign of the linear term, suggesting that the slope of the exposure- effect curve was for most observed associations weaker at high PFAS serum concentrations than at low concentrations.

The results of the continuous piecewise linear regressions for 5 of the most relevant PFAS parameters are shown in supplementary materials Table S3. Due to the limited number of subject in each of the six concentration ranges studied the regression coefficients are too variable to quantify the course of the exposure-effect curves in greater detail in a meaningful way. However, for the 39 associations studied with piecewise regression, the strongest regression coefficient was observed 36 times among the concentration ranges below the median PFAS serum concentration, and only 3 times among the higher concentration ranges suggesting that the slope of the studied associations was steeper below median PFAS concentrations. The strongest regression coefficient was found respectively 12, 14 and 10 times in the “< p10”, ”p10 to< p25”, “p25 to< p50”,ranges and only 1, 2 and zero times in the “p50 to< p75”, “p75 to < p 90” and “p90 and higher” ranges.

## Discussion

A fully referenced version of this discussion is included in the supplementary materials.

In our study on the associations of internal exposure to PFAS with thyroid and sex hormone concentrations and pubertal development, the strength of the association was, consistent with our work hypothesis, in most cases clearly much stronger below the corresponding median PFAS serum concentration than above. For the sum of PFNA and linear PFHxS, PFOS, and PFOA the median serum concentration for adolescents in our study amounted to 4.40 µg/L, for PFOS to 2.4 µg/L and for PFOA to 1.1 µg/L. EFSA has defined a normative health-based guidance value of a tolerable weekly intake (TWI) of 4.4 nanograms per kilogram of body weight per week for the sum of PFNA and linear PFHxS, PFOS, and PFOA (https://www.efsa.europa.eu/en/news/pfas-food-efsa-assesses-risks-and-sets-tolerable-intake). This EFSA norm corresponds to 6.9µG/L serum for the sum of PFNA and linear PFHxS, PFOS, and PFOA. The most strict HBMI reference serum concentration values defined by the German Human Biomonitoring Commission are 5 µg/L for PFOS and 2 µg/L for PFOA (HBM commission, 2016).

Detailed data on exposure-response relations concerning PFAS effects on humans are limited but indicate the occurrence of complex non-monotonic exposure-response curves.

Although, in our study, the regression coefficients for associations limited to PFAS values below the median were often quite strong, statistical significance was often not reached. The lack of statistical significance can be explained by the small number of subjects and also by the limited precision of the determination of the PFAS concentrations at low concentrations such as those below the median values. It can be expected that, for technical reasons, the measurement of PFAS compounds in serum at concentrations below the observed median values is fraught with some uncertainties. That the concentrations below the median were indeed measured with less precision than those above the median is suggested by the fact that the standard errors on and 95% confidence limit widths of the regression coefficients concerning associations with PFAS concentrations below median values were much larger than those concerning PFAS concentrations above median values (see Table 3). Additionally, in a study limited to individuals for whom the sum of the serum concentrations of PFHxS(total), PFOA(total), PFOS(total), PFDA and PFNA was above the corresponding median serum concentration, the correlations between the different PFAS congeners were consistently positive and significant in 13 of the 15 correlations examined (see supplementary material Table S4). In contrast, in a study limited to individuals for whom the same sum of PFAS values was below the corresponding median serum concentration, 5 of the 15 correlations between congeners examined were negative, 9 of the 10 positive correlations were significant, and 7 of the 10 positive correlations were weaker than the corresponding correlations above the median (see supplementary Table S4). The fact that the correlation between different PFAS compounds is weaker at concentrations below the median than above the median (see supplementary material Table S4) also points to less precise measurements below the median serum concentrations. In studies seeking to establish a exposure-response relationship, inaccuracies in determining exposure will systematically lead to an underestimation of the increase in risk [3]. The lack of precision in the PFAS determinations below the median serum concentrations may contribute to an underestimation of the actual differences in regression coefficients below and above the median and contributes, together with the decrease in number of observations, to the fact that, in spite of often strong regression coefficients, far less significant associations were found at PFAS concentrations below the median than described by van Larebeke et al. (2025) [1].

That lower PFAS serum concentrations have per unit of exposure (µg/L) stronger effects on certain biological or health parameters can be explained by and is probably due to the fact that these effects are induced by the interaction of PFAS with receptors. Our present finding shares some characteristics with dose-response curves that are quite frequently observed for some other endocrine disrupting chemicals showing stronger associations at lower doses than at higher doses. The mechanisms through which PFAS act include binding to different receptors as reported in many publications. PFAS thus act as receptor binding endocrine disrupting substances. Natural hormones and receptor-binding endocrine disrupting chemicals such as TCDD tend to display, in arithmetical plots, in relation with primary effects such as changes in gene expression, an asymptotic-like dose-response curve with an almost linear steep response at very low doses, followed by saturation at higher concentrations reflected in exposure-effect curves which are almost horizontal [2]. TCDD has many biological and health effects, most of which are based on the interaction with the AH receptor (AhR), and primary responses, such as the induction of the expression of CYP I A1 and IA2 show a asymptotic-like dose response curve [4]. More complex effects can show different dose-response curves as other signal transduction pathways and tissue- and cell-specific factors can modulate the qualitative and quantitative relationship between receptor occupancy and response [4]. But dose-response curves for effects associated with dioxin exposure often show stronger associations (stronger regression coefficients) at low exposures than at higher exposures. This has been observe for three non-sparsely occurring reproductive outcomes, including not live born, miscarriage, and preterm in a study on male Air Force veterans of the Vietnam War [5]. Also, in a National Health and Nutrition Examination Survey (NHANES) study on 2,992 persons’ a positive linear exposure-effect relation was observed between the log10 transformed serum concentration of 1,2,3,4,6,7,8-heptachlorodibenzofuran and the increase in risk of cancer mortality, implying that per pg/g blood fat the increase in cancer risk was higher at low exposures than at high exposures [6].

Also for Bisphenol A, one of the most studied endocrine disrupting chemicals, low doses were observed to cause more intense effects than higher doses: the variation of ductal thickness in the mammary gland of rats, indicating an increase in budding, increased between 0 and 25 µg/kg body weight /day, then dropped, with a breaking point between 25 and 250 µg/kg body weight /day, to increase again at doses between 250 and 25,000 µg/kg body weight /day [7]. In Cancer tests on the same cohort of rats, Bisphenol A induced a statistically significant increased incidence of adenocarcinoma, increase that was not observed at higher doses [8]. Interestingly, Villar-Pazos et al. (2017) [9] showed that BPA dose-response curves can be monotonic for some end points and non-monotonic for other endpoints.

That endocrine disrupting PFAS effects might be, per µg/L serum of internal exposure, relatively more pronounced at low internal exposures than at high internal exposures is consistent with the observation reported in many publications that occupational or severe environmental exposures up to more than a hundred times higher than the environmental exposures observed in general populations, are associated with health effects that are certainly serious but not a hundred times worse than those observed in general populations.

Probably the endocrine disrupting effects such as those observed in our studies are due to interactions with receptors as these have been documented in many publications. However, PFAS interfere with many other biological mechanisms as reported in the literature. They cause oxidative stress, infiltrate lipid membranes, adhere to proteins, affect DNA methylation, affect acetylation of histones, inhibit of gap junctional intercellular communication and increase telomere length. So it is likely that they might have other biological and health effects with exposure-effect relationships different from those discussed here.

The limitations acknowledged in the “original study” also affect the present study. These concern the number of subjects, the cross-sectional nature, the choice not to adjust for dietary factors, the lack of information about the iodine status of the participants, the use of single-pollutant models not taking into account mixture effects, the assessment of stage of pubertal development resting on self-administered questionnaires. These limitations were extensively discussed by van Larebeke et al. (2025) [1]. In addition the statistical method to account for the clustering within households (there were 43 households with 2 adolescents participating in the study on the total of 303 adolescents originally recruited for the study) was not available for the present study.

The strengths of the “original study” concerning validated chemical analysis, validated clinical biological analysis and sampling and biological measurements performed by trained study nurses also sustain the present study. Many statistical associations were tested, increasing the likelihood of chance findings. However, in accordance with the views of the epidemiologist Kenneth Rothman in his book “Modern epidemiology” (ISBN 0-316-75776-4) and in view of the known endocrine disrupting properties of PFAS, which render observed exposure-effect relations biologically plausible, we did not apply corrections for multiple testing. The way in which testing for statistical significance is done and interpreted can, in some circumstances, prevent important biological/health data or mechanisms from being observed as explained by Kenneth Rothman in “Moden Epidemiology” and in a later publication [10].

## Conclusions

Our observations show that, in our study on adolescents, the associations between PFAS internal exposure and biologically relevant differences in blood hormone levels and pubertal development were for most parameters clearly stronger below the median PFAS serum concentrations observed in those adolescents than above these median concentrations, although these median concentrations were below the range of the health based guidance values below which no adverse health effects are expected. Our observations suggest that an important part of the endocrine disrupting effect of PFAS on adolescents occurs at serum levels below present health based guidance values.

## Supplementary Information

Supplementary Material 1.

## Data Availability

Access to data and materials can be requested from the “Toezichtcommissie van de Vlaamse Humane biomonitoring (Monitoring committee of the Flemish Human Biomonitoring). This committee can be contacted through [TZC@provincieantwerpen.be](mailto: TZC@provincieantwerpen.be).

## Abbreviations

4PFAS: Sum of the linear PFOA, PFNA, PFHxs, and PFOS
7PFAS: Sum of linear N-Methylperfluorooctanesulfonamide (MePFOSAA), linear PFHxS, total PFOA, linear PFDA, linear Perfluoroundecanoic acid (PFUnDA), total PFOS, and linear PFNA
BELAC: Belgian accreditation system
EFSA: The European Food Safety Authority
FT3: Free triiodothyronine
lb4PFAS: Sum of total PFOA, linear PFNA, total PFHxS, and total PFOS
LH: Luteinising hormone
LOD: Limit of detection
LOQ: Limit of quantification
MePFOSAA: N-Methylperfluorooctanesulfonamide
NHANES: National Health and Nutrition Examination Survey
OR: Odds ratio
P4: Progesterone
PDS: Pubertal development scale
PFAS: Per- and polyfluoroalkyl substances
PFBA: Perfluorobutanoic acid
PFDA: Perfluorodecanoic Acid
PFHxS: Perfluorohexanesulfonic acid
PFNA: Perfluorononanoic acid
PFOA: Perfluorooctanoic acid
PFOS: Perfluorooctanesulfonic acid
SHBG: Sex hormone-binding globuli
TSH: Thyroid stimulating hormone
T3: Triiodothyronine
Vs: Versus (against)
