# Supplementary material for: PFAS endocrine disruption affecting pubertal development: exposure-response is steeper below median PFAS serum concentrations
Source: Environ Health. 2026 Mar 12;25:37. doi: 10.1186/s12940-026-01285-9 (PMC13093952; doi:10.1186/s12940-026-01285-9)
Supplement: Supplementary file 1 — Supplementary Material 1. [file 12940_2026_1285_MOESM1_ESM.docx]

**Supplementary materials to**

**PFAS endocrine disruption affecting pubertal development: exposure-response is steeper below median PFAS serum concentrations** by van Larebeke et al.

**List of contents of Supplementary materials**

| Type of information | Page |
| --- | --- |
| Additional references that might be interesting to the reader | 3 |
| Table S1 | 4 |
| Table S2 | 7 |
| Table S3 | 12 |
| Table S4 | 16 |
| Figure S1 . Histogram of length of male adolescents | 17 |
| Figure S2 Histogram of TSH serum concentrations in male adolescents | 18 |
| Figure S3. Histogram of FT3 serum concentrations in male adolescents | 19 |
| Figure S4. Histogram of FT3 serum concentrations in female adolescents | 20 |
| Figure S5. Histogram of LH concentrations in male adolescents | 21 |
| Figure S6. Histogram of total testosterone concentrations in male adolescents | 22 |
| Figure S7. Histogram of bioavailable testosterone concentrations in male adolescents | 23 |
| Figure S8. Histogram of SHBG concentrations in male adolescents | 24 |
| S9. Female adolescents. Association between serum concentration of PFOA and proportional Odds ratio for breast development for PFOA concentrations below the median | 25 |
| S10. Female adolescents. Association between serum concentration of PFOA and proportional Odds ratio for breast development for PFOA concentrations above the median | 26 |
| S11. Female adolescents. Association between serum concentration of PFOA and proportional Odds ratio for breast development for PFOA concentrations below the median on a scale permitting comparison with the graph concerning the association for PFOA concentrations above the median | 27 |
| S12. Female adolescents. Association between serum concentration of PFOA and proportional Odds ratio for breast development for PFOA concentrations above the median on a scale permitting comparison with the graph concerning the association for PFHxS concentrations below the median | 28 |
| Figures S13. A series of 67 Distance Weighted Least Square Graphs, one for each of the significant associations concerning male adolescents reported in the “original study” by van Larebeke et al. (2025) (ref 1 in this manuscript). | 29-90 |
| Figures S14. A series of 17 Distance Weighted Least Square Graphs, one for each of the significant associations concerning female adolescents reported in the “original study” by van Larebeke et al.(2025) (ref 1 in this manuscript). | 91-105 |
| Fully referenced version of the Discussion | 106-111 |

**Additional references that might be interesting to the reader**

*Per- and polyfluoroalkyl substances (PFAS) are omnipresent in the environment and in human beings and can remain a long time in the human body, with half-lives of up to 8.5 years (Olsen et al., 2007).*

Olsen GW, Burris JM, Ehresman DJ, Froehlich JW, Seacat AM, Butenhoff JL, Zobel LR (2007) Half-life of serum elimination of perfluorooctanesulfonate, perfluorohexanesulfonate, and perfluorooctanoate in retired fluorochemical production workers. Environ Health Perspect 115:1298–1305

**Table S1. Results from sensitivity analysis performed on the 9 associations showing a stronger slope below the median and involving PFOS or 4PFAS, the PFAS parameters for which the most extreme values were found**

| Effect^a^ | PFAS µg/L^b^ | Sign^c^ | Above median^d^ | | | | | Above median^d^ after exclusion of values equal or superior to the 97 percentile | | | | |
| --- | --- | --- | --- | --- | --- | --- | --- | --- | --- | --- | --- | --- |
|  |  |  | Regression coefficient  (odds ratio if relevant) | Standard  error | Regression coefficient lower 95 % CI  (odds ratio if relevant) | Regression coefficient upper 95%CI  (odds ratio if relevant) | p | Regression coefficient | Standard error | Regression coefficient lower 95 % CI  (odds ratio if relevant) | Regression coefficient upper 95 % CI  (odds ratio if relevant) | p |
| Observations on male adolescents | | | | | | | | | | | | |
| SHBG nmol/L | PFOS | Pos. | 0.10 | 0.14 | -0.18 | 0.38 | 0.49 | -0,0525 | 0,25068 | -0,5438 | 0,4388 | 0,83 |
| LH IU/L | PFOS | Neg. | -0.009 | 0.006 | -0.021 | 0.003 | 0.16 | 0,0069 | 0,01060 | -0,0139 | 0,0277 | 0,51 |
| Latent score^d^ in binary logit model for Progestrone above limit of detection | PFOS | Neg. | -0.0067 (0.993) | 0.0073 | -0.0209 (0.979) | 0.0076 (1.008) | *0.36* | 0,0164 (1.015) | 0,0198 (1.019) | -0,022 (0.978) | 0,0553 (1.057) | 0,40 |
| Latent OMP score^e^ for Body Hair growth | PFOS | Neg. | -0.005 | 0.010 | -0.024 | 0.014 | 0.60 | -0,0349 | 0,4151 | -0,8484 | 0,7787 | 0,93 |
| Bioavailable testosteron ng/dL | 4PFAS | Neg. | -0.54 | 0.49 | -1.51 | 0.43 | 0.28 | 0,172 | 0,819 | -1,434 | 1,778 | 0,83 |
| SHBG nmol/L | 4PFAS | Pos. | 0.14 | 0.13 | -0.12 | 0.39 | 0.30 | 0,0338 | 0,2247 | -0,4066 | 0,4742 | 0,88 |
| LH IU/L | 4PFAS | Neg. | -0.007 | 0.006 | -0.019 | 0.004 | 0.22 | 0,0063 | 0,0101 | -0,0135 | 0,0261 | 0,53 |
| Latent OMP score^f^for Growth Spurt | 4PFAS | Neg. | -0.004 | 0.009 | -0.021 | 0.014 | 0.69 | 0,0048 | 0,0150 | -0,0246 | 0,0342 | 0,75 |
| Latent OMP score^e^ for Body Hair growth | 4PFAS | Neg. | -0.0072 | 0.0090 | -0.0249 | 0.0105 | 0.40 | -0,0057 | 0,0147 | -0,0345 | 0,0232 | 0,70 |

*^a^Effect parameter as used in van Larebeke et al.(2025)*

*^b^Parameter of internal exposure as used in van Larebeke et al.(2025)*

*^c^Sign of the statistically significant association as described in van Larebeke et al.(2025). Negative if higher PFAS serum concentrations are associated with lower values of the effect parameter.*

^d^The change in the latent score in a Binomial logit model per increase of 1 µg/L PFAS is the regression coefficient for the effect of the PFAS concentration on reaching a level of progesterone above the limit of detection

^e^ The change in the latent score in an Ordinal Multinomial Probit model per increase of 1 µg/L PFAS is the regression coefficient for the effect of the PFAS concentration on reaching a further stage of body hair growth

^f^The change in the latent score in an Ordinal Multinomial Probit model per increase of 1 µg/L PFAS is the regression coefficient for the effect of the PFAS concentration on reaching a further stage of growth spurt

**Table S2. Comparison between linear and quadratic terms in multiple regressions concerning associations described by van Larebeke et al. (2025)**

| Effect^a^ | PFAS µg/L^b^ | *Sign^c^* | *Linear term^d^* | | | | | *Quadratic term^d^ ^e^* | | | | |
| --- | --- | --- | --- | --- | --- | --- | --- | --- | --- | --- | --- | --- |
|  |  |  | *Regression coefficient* | *Standard*  *error* | *Regression coefficient lower 95 % CI* | *Regression coefficient upper 95%CI* | *p* | *Regression coefficient* | *Standard error* | *Regression coefficient lower 95 % CI* | *Regression coefficient upper 95 % CI* | *p* |
| Observations in male adolescents | | | | | | | | | | | | |
| Height cm | PFOA | Neg. | -6,14 | 2,48 | -11,00 | -1,29 | 0,013 | 0,87 | 0,50 | -0,11 | 1,85 | 0,080 |
| Height cm | Lb4PFAS | Neg. | -0,179 | 0,104 | -0,383 | 0,025 | 0,086 | 0,00103 | 0,00116 | -0,00124 | 0,00330 | 0,37 |
| FT3 ng/dL | PFOA | Pos | 0,011 | 0,010 | -0,008 | 0,031 | 0,25 | 0,0003 | 0,0020 | -0,0036 | 0,0042 | 0,88 |
| FT3 ng/dL | PFHxS | Pos | 0,021 | 0,011 | -0,00007 | 0,0420 | 0,051 | -0,0044 | 0,0033 | -0,0108 | 0,0021 | 0,19 |
| Totaal Testosteron ng/dL | PFOA | Neg. | -143,6 | 63,20 | -267,5 | -19,7 | 0,023 | 22,5 | 12,7 | -2,39 | 47,4 | 0,076 |
| Totaal Testosteron ng/dL | PFHxS | Neg. | -140,3 | 67,3 | -272,2 | -8,34 | 0,037 | 24,5 | 20,7 | -16,0 | 65,0 | 0,24 |
| Bioavailable testosteron ng/dL | PFOA | Neg. | -84,2 | 27,1 | -137,3 | -30,99 | 0,002 | 13,1 | 5,46 | 2,41 | 23,80 | 0,016 |
| Bioavailable testosteron ng/dL | PFHxS | Neg. | -74,2 | 29,0 | -131,0 | -17,3 | 0,011 | 12,7 | 8,91 | -4,78 | 30,13 | 0,15 |
| Bioavailable testosteron ng/dL | PFOS | Neg. | -1,19 | 1,36 | -3,86 | 1,48 | 0,38 | 0,0023 | 0,0198 | -0,036 | 0,041 | 0,91 |
| Bioavailable testosteron ng/dL | 4PFAS | Neg. | -1,52 | 1,33 | -4,13 | 1,09 | 0,25 | 0,0065 | 0,0179 | -0,029 | 0,042 | 0,72 |
| Bioavailable testosteron ng/dL | Lb4PFAS | Neg. | -1,42 | 1,16 | -3,70 | 0,85 | 0,22 | 0,0052 | 0,0129 | -0,0201 | 0,0304 | 0,69 |
| SHBG nmol/L | PFHxS | Pos. | 10,7 | 6,54 | -2,09 | 23,56 | 0,10 | -1,60 | 2,01 | -5,54 | 2,33 | 0,42 |
| SHBG nmol/L | PFOS | Pos. | 0,254 | 0,302 | -0,339 | 0,846 | 0,40 | -0,00063 | 0,00438 | -0,0092 | 0,0080 | 0,89 |
| SHBG nmol/L | 4PFAS | Pos. | 0,300 | 0,296 | -0,280 | 0,879 | 0,31 | -0,0012 | 0,0040 | -0,0090 | 0,0066 | 0,77 |
| LH IU/L | PFOA | Neg. | -0,993 | 0,371 | -1,72 | -0,27 | 0,007 | 0,158 | 0,075 | 0,012 | 0,304 | 0,034 |
| LH IU/L | PFHxS | Neg. | -0,456 | 0,400 | -1,24 | 0,33 | 0,25 | 0,020 | 0,123 | -0,221 | 0,261 | 0,87 |
| LH IU/L | PFOS | Neg. | -0,0118 | 0,0185 | -0,048 | 0,024 | 0,52 | -0,00004 | 0,00027 | -0,00056 | 0,00048 | 0,88 |
| LH IU/L | 4PFAS | Neg. | -0,0156 | 0,0181 | -0,0510 | 0,0198 | 0,39 | 0,000017 | 0,000242 | -0,00046 | 0,00049 | 0,94 |
| LH IU/L | Lb4PAS | Neg. | -0,0210 | 0,0157 | -0,0517 | 0,0097 | 0,18 | 0,00008 | 0,00017 | -0,00026 | 0,00042 | 0,64 |
| Latent score^e^ in binary logit model for Progestrone above limit of detection | PFOS | Neg. | 0,0046 | 0,0214 | -0,0373 | 0,0465 | 0,83 | -0,00015 | 0,00027 | -0,00069 | 0,000383 | 0,58 |
| Latent score^e^ in binary logit model for Progestrone above limit of detection | 4PFAS | Neg. | 0,0153 | 0,0220 | -0,0279 | 0,0585 | 0,49 | -0,00025 | 0,00027 | -0,00078 | 0,00027 | 0,35 |
| Latent score^e^ in binary logit model for Progestrone above limit of detection | Lb4PAS | Neg. | 0,0232 | 0,0200 | -0,0159 | 0,0624 | 0,24 | -0,00027 | 0,00021 | -0,00068 | 0,00014 | 0,19 |
| Latent OMP score^f^ for Growth Spurt | PFOA | Neg | -1,51 | 0,45 | -2,39 | -0,64 | 0,0007 | 0,22 | 0,090 | 0,042 | 0,393 | 0,015 |
| Latent OMP score^f^ for Growth Spurt | PFHxS | Neg. | -1,54 | 0,475 | -2,47 | -0,613 | 0,001 | 0,327 | 0,142 | 0,049 | 0,606 | 0,021 |
| Latent OMP score^f^ for Growth Spurt | PFOS | Neg. | -0,022 | 0,020 | -0,061 | 0,018 | 0,29 | 0,00013 | 0,00029 | -0,00044 | 0,00071 | 0,65 |
| Latent OMP score^f^ for Growth Spurt | 4PFAS | Neg. | -0,029 | 0,020 | -0,068 | 0,010 | 0,14 | 0,00022 | 0,00027 | -0,00030 | 0,00074 | 0,41 |
| Latent OMP score^f^ for Growth Spurt | Lb4PFAS | Neg. | -0,024 | 0,018 | -0,059 | 0,010 | 0,17 | 0,00014 | 0,00020 | -0,00024 | 0,00053 | 0,46 |
| Latent OMP score^g^ for Body Hair growth | PFHxS | Neg | -1,31 | 0,47 | -2,23 | -0,39 | 0,005 | 0,313 | 0,143 | 0,033 | 0,594 | 0,029 |
| Latent OMP score^g^ for Body Hair growth | PFOS | Neg | -0,044 | 0,021 | -0,086 | -0,002 | 0,040 | 0,00045 | 0,00031 | -0,00016 | 0,00105 | 0,15 |
| Latent OMP score^g^ for Body Hair growth | 4PFAS | Neg. | -0,043 | 0,021 | -0,084 | -0,002 | 0,039 | 0,00041 | 0,00028 | -0,00014 | 0,00095 | 0,15 |
| Latent OMP score^g^ for Body Hair growth | Lb4PFAS | Neg | -0,036 | 0,018 | -0,0722 | -0,0005 | 0,047 | 0,00027 | 0,00020 | -0,00013 | 0,00067 | 0,19 |
| Observations in female adolescents | | | | | | | | | | | | |
| FT3 ng/dL | PFOS(branched) | Neg. |  |  |  |  |  |  |  |  |  |  |
| Latent score^h^ in binary logit model for reaching Pubertal Stage expected for age | PFOA | Neg. | -1,96 | 2,41 | -6,69 | 2,78 | 0,42 | 0,206 | 0,934 | -1,62 | 2,04 | 0,83 |
| Latent score^h^ in binary logit model for reaching Pubertal Stage expected for age | PFHxS | Neg. | -0,402 | 0,590 | -1,56 | 0,75 | 0,50 | -0,0042 | 0,1390 | -0,277 | 0,268 | 0,98 |
| Latent score^h^ in binary logit model for reaching Pubertal Stage expected for age | PFOS | Neg. | -0,0122 | 0,0206 | -0,0526 | 0,0283 | 0,56 | -0,000005 | 0,000173 | -0,00034 | 0,00033 | 0,98 |
| Latent score^h^ in binary logit model for reaching Pubertal Stage expected for age | 4PFAS | Neg. | -0,0136 | 0,0197 | -0,0522 | 0,0250 | 0,49 | 0,000007 | 0,000148 | -0,00028 | 0,00030 | 0,96 |
| Latent OMP score^f^  for Growth Spurt | PFOA | Neg. | -2,51 | 1,39 | -5,24 | 0,22 | 0,072 | 0,759 | 0,521 | -0,26 | 1,78 | 0,14 |
| Latent OMP score^g^ for Body Hair growth | PFOA | Neg. | -2,12 | 1,23 | -4,52 | 0,28 | 0,084 | 0,598 | 0,459 | -0,301 | 1,498 | 0,19 |
| Latent OMP score^g^ for Body Hair growth | PFHxS | Neg. | -0,289 | 0,226 | -0,731 | 0,153 | 0,20 | 0,016 | 0,031 | -0,045 | 0,077 | 0,60 |
| Latent OMP score^i^ for Breast Development | PFOA | Neg. | -4,47 | 1,34 | -7,09 | -1,849 | 0,0008 | 1,39 | 0,493 | 0,42 | 2,356 | 0,0049 |

*^a^Effect parameter as used in van Larebeke et al.(2025)*

*^b^Parameter of internal exposure as used in van Larebeke et al.(2025)*

*^c^Sign of the statistically significant association as described in van Larebeke et al.(2025). Negative if higher PFAS serum concentrations are associated with lower values of the effect parameter.*

^d^ Regression coefficients were derived from multiple regressions incorporating a term for the PFAS concentration, a term for (PFAS concentration)^2^ and adjustment for age (except in relation with the parameter “Pubertal Stage expected for age”) and for the binary variable “making ends meet with the income– manage to live comfortably”. Data related to a p value<0.05 are marked in red, data related to a p value <0.1 are marked in pink.

^e^The change in the latent score in a Binomial logit model per increase of 1 µg/L PFAS is the regression coefficient for the effect of the PFAS concentration on reaching a level of progesterone above the limit of detection

^f^The change in the latent score in an Ordinal Multinomial Probit model per increase of 1 µg/L PFAS is the regression coefficient for the effect of the PFAS concentration on reaching a further stage of growth spurt

^g^ The change in the latent score in an Ordinal Multinomial Probit model per increase of 1 µg/L PFAS is the regression coefficient for the effect of the PFAS concentration on reaching a further stage of body hair growth

^h^ The change in the latent score in a Binomial logit model per increase of 1 µg/L PFAS is the regression coefficient for the effect of the PFAS concentration on reaching the Pubertal stage expected for age

^i^ The change in the latent score in an Ordinal Multinomial Probit model per increase of 1 µg/L PFAS is the regression coefficient for the effect of the PFAS concentration on reaching a further stage of breast development

The quadratic term was found to be significant in six and marginally significant in two of the 39 studied regressions entailing both a linear and quadratic term for PFAS concentration, indicating in those associations the existence of non-linearity. In 35 of the 39 studied regressions the quadratic term had a sign opposed to the sign of the linear term, suggesting that the slope of the dose effect curve was for most observed associations weaker at high PFAS serum concentrations than at low concentrations.

**Table S3.Regression coefficients determined through continuous piecewise regression for six successive PFAS serum concentration ranges**

| PFAS | Effect parameter | Sign in original study | Regression coefficients related to PFAS serum concentration ranges defined below^d^ | | | | | |
| --- | --- | --- | --- | --- | --- | --- | --- | --- |
|  |  |  | Under p10^d^ | P10 to<p25^d^ | P25 to<p50^d^ | P50 to<p75^d^ | P75 to <p90^d^ | P90^d^ and above |
| Observations on male adolescents | | | | | | | | |
| PFOA | Height cm | Neg. | -8,17 | 15,04 | -14,46 | -2,45 | 11,94 | -2,66 |
| PFOA | FT3 ng/dL | Pos. | 0,0015 | 0,0464 | 0,0428 | -0,1576 | 0,1021 | -0,0228 |
| PFOA | Total Testosteron ng/dL | Neg. | -98,4 | 207,9 | -464,8 | 338,5 | -74,5 | 104,7 |
| PFOA | Bioavailable testosteron ng/dL | Neg. | -38,8 | -113,3 | 35,2 | 162,7 | -145,2 | 106,9 |
| PFOA | Luteinizing hormone IU/L | Neg. | -0,202 | -2,970 | 3,647 | -1,731 | 1,025 | 0,302 |
| PFOA | Latent OMP score^e^ for Growth Spurt | Neg. | 1,266 | -4,060 | -0,272 | 1,636 | 1,448 | -0,107 |
| PFHxS | FT3 ng/dL | Pos | 0,147 | 0,138 | -0,330 | 0,016 | 0,073 | -0,055 |
| PFHxS | Total Testosteron ng/dL | Neg | -1665 | 3341 | -2900 | 1181 | 157 | -200 |
| PFHxS | Bioavailable testosteron ng/dL | Neg | -825,5 | 1063,6 | -658,2 | 385,4 | 72,1 | -74,0 |
| PFHxS | sex hormone-binding globulin (nmol/L) | Pos | 32,15 | 0,67 | -7,52 | -30,91 | 19,04 | -13,54 |
| PFHxS | Luteinizing hormone IU/L | Neg | 3,28 | -3,00 | -7,16 | 6,88 | 0,81 | -1,53 |
| PFHxS | Latent OMP score^e^ for Growth Spurt | Neg | -12,78 | 21,96 | -15,76 | 5,00 | 1,92 | -0,35 |
| PFHxS | Latent OMP score^f^ for Body Hair growth | Neg | -7,44 | -4,61 | 14,41 | -4,36 | 1,99 | 0,17 |
| PFOS | Bioavailable testosteron ng/dL | Neg. | 53,62 | -49,98 | -15,61 | -1,12 | 16,14 | -4,47 |
| PFOS | sex hormone-binding globulin (nmol/L) | Pos. | 58,46 | -73,61 | 15,01 | 4,17 | -4,55 | 0,68 |
| PFOS | Luteinizing hormone IU/L | Neg. | 0,094 | -1,770 | 1,824 | -0,357 | 0,274 | -0,090 |
| PFOS | Latent score^g^ in binary logit model for Progestrone above limit of detection | Neg. | -0,140 | 1,120 | -1,766 | 0,970 | -0,195 | 0,001 |
| PFOS | Latent OMP score ^e^ for Growth Spurt | Neg. | -0,791 | 0,993 | -0,222 | -0,153 | 0,187 | -0,024 |
| PFOS | Latent OMP score^f^ for Body Hair growth | Neg | 0,637 | -1,789 | 0,870 | 0,190 | 0,084 | 0,006 |
| 4PFAS | Bioavailable testosteron ng/dL | Neg. | -6,48 | -26,97 | 35,53 | -13,83 | 14,40 | -3,94 |
| 4PFAS | sex hormone-binding globulin (nmol/L) | Pos. | 16,02 | -11,95 | -7,78 | 6,76 | -3,30 | 0,37 |
| 4PFAS | Luteinizing hormone IU/L | Neg. | -0,128 | -0,416 | 0,392 | 0,023 | 0,184 | -0,079 |
| 4PFAS | Latent score^g^ in binary logit model for Progestrone above limit of detection | Neg | 0,468 | -0,265 | -0,432 | 0,353 | -0,148 | 0,013 |
| 4PFAS | Latent OMP score^e^ for Growth Spurt | Neg. | 0,625 | -1,722 | 1,087 | -0,102 | 0,124 | -0,022 |
| 4PFAS | Latent OMP score^f^ for Body Hair growth | Neg | -0,014 | -1,101 | 1,126 | -0,042 | 0,000 | 0,032 |
| Lb4PFAS | Height cm | Neg. | -2,400 | 3,178 | -2,168 | 1,337 | 0,096 | -0,126 |
| Lb4PFAS | Bioavailable testosteron ng/dL | Neg. | -22,99 | 21,81 | -5,39 | -0,93 | 11,29 | -5,47 |
| Lb4PFAS | Luteinizing hormone IU/L | Neg. | -0,451 | 0,537 | -0,142 | -0,055 | 0,151 | -0,060 |
| Lb4PFAS | Latent score^g^ in binary logit model for Progestrone above limit of detection | Neg. | 0,117 | 0,109 | -0,381 | 0,252 | -0,105 | -0,003 |
| Lb4PFAS | Latent OMP score^e^ for Growth Spurt | Neg. | -0,207 | -0,058 | 0,297 | -0,169 | 0,177 | -0,055 |
| Lb4PFAS | Latent OMP score^f^ for Body Hair growth | Neg | -0,228 | 0,248 | -0,131 | 0,054 | 0,055 | -0,003 |
| Observations on female adolescents | | | | | | | | |
| PFOA | Latent OMP score^e^ for Growth Spurt | Neg | -1,96 | 0,86 | 1,89 | -2,91 | 1,42 | 2,77 |
| PFOA | Latent score^h^ in binary logit model for reaching Pubertal Stage expected for age | Neg. | 1,79 | -7,93 | 4,13 | 3,19 | -4,45 | 2,27 |
| PFOA | Latent OMP score^f^ for Body Hair growth | Neg | 0,85 | -4,83 | 5,57 | -4,05 | 2,24 | 1,41 |
| PFOA | Latent OMP score^i^ for Breast Development B | Neg | -1,38 | -0,79 | -1,67 | 4,10 | -0,12 | 0,54 |
| PFHxS | Latent score^h^ in binary logit model for reaching Pubertal Stage expected for age | Neg | -7,03 | 14,72 | -24,83 | 23,94 | -8,64 | 1,66 |
| PFHxS | Latent OMP score^f^ for Body Hair growth | Neg | 1,46 | -6,48 | 1,79 | 2,13 | 1,84 | -0,88 |
| PFOS | Latent score^h^ in binary logit model for reaching Pubertal Stage expected for age | Neg. | -4,27 | 6,31 | -2,54 | 0,47 | -0,01 | 0,03 |
| 4PFAS | Latent score^h^in binary logit model for reaching Pubertal Stage expected for age | Neg. | 0,253 | -0,889 | 0,366 | 0,220 | 0,031 | 0,015 |

*^a^Parameter of internal exposure as used in van Larebeke et al.(2025)*

*^b^Effect parameter as used in van Larebeke et al.(2025)*

*^c^Sign of the statistically significant association as described in van Larebeke et al.(2025). Negative if higher PFAS serum concentrations are associated with lower values of the effect parameter.*

*^d^ For each of the PFAS parameters the regression coefficients were calculated for the concentration ranges bounded by the percentiles p10, p25, p50, p75 and p90 through a continuous piecewise linear regression. The strongest observed regression coefficient is marked in red.*

^e^ The change in the latent score in an Ordinal Multinomial Probit model per increase of 1 µg/L PFAS is the regression coefficient for the effect of the PFAS concentration on reaching a further stage of growth spurt

^f^ The change in the latent score in an Ordinal Multinomial Probit model per increase of 1 µg/L PFAS is the regression coefficient for the effect of the PFAS concentration on reaching a further stage of body hair growth

^g^The change in the latent score in a Binomial logit model per increase of 1 µg/L PFAS is the regression coefficient for the effect of the PFAS concentration on reaching a level of progesterone above the limit of detection

^h^The change in the latent score in a Binomial logit model per increase of 1 µg/L PFAS is the regression coefficient for the effect of the PFAS concentration on reaching the Pubertal stage expected for age

^i^The change in the latent score in an Ordinal Multinomial Probit model per increase of 1 µg/L PFAS is the regression coefficient for the effect of the PFAS concentration on reaching a further stage of breast development

**Table S4. Correlations between PFAS compounds amongst on the one hand adolescents having for the sum of PFHxS(total), PFOA(total), PFOS(total), PFDA and PFNA a serum concentration below the median value and on the other adolescents having for this sum of PFAS compounds a serum concentration above the median**

| Correlation between | For adolescents having for the sum of PFHxS(total),PFOA(total) PFOS(total) PFDA and PFNA a serum concentration below the median | For adolescents having for the sum of PFHxS(total),PFOA(total) PFOS(total) PFDA and PFNA a serum concentration above the median |
| --- | --- | --- |
| PFOA(total) and PFOS | 0,248636 | 0,282939 |
| PFOA(total) and PFOS(branched) | -0,126234 | 0,100169 |
| PFOA(total) and PFNA | 0,485718 | 0,481569 |
| PFOA(total) and PFDA | 0,462330 | 0,562206 |
| PFHxS(total) and PFOS | 0,287389 | 0,791047 |
| PFHxS(total) and PFOS(branched) | -0,214863 | 0,194466 |
| PFHxS(total) and PFNA | 0,284341 | 0,312330 |
| PFHxS(total) and PFDA | 0,049720 | 0,247647 |
| PFOS and PFOS(branched) | -0,125655 | 0,274571 |
| PFOS and PFNA | 0,441068 | 0,374192 |
| PFOS and PFDA | 0,206569 | 0,275561 |
| PFOS(branched) and PFNA | -0,009429 | 0,182041 |
| PFOS(branched) and PFDA | -0,031282 | 0,053593 |
| PFNA and PFDA | 0,565093 | 0,809447 |

**Supplementary materials Figures**

**Figure S1. Histogram of length of male adolescents**

**Figure S2. Histogram of TSH serum concentrations in male adolescents**

**Figure S3. Histogram of FT3 serum concentrations in male adolescents**

**Figure S4. Histogram of FT3 serum concentrations in female adolescents**

**Figure S5. Histogram of LH concentrations in male adolescents**

**Figure S6. Histogram of total testosterone concentrations in male adolescents**

**Figure S7. Histogram of bioavailable testosterone concentrations in male adolescents**

**Figure S8. Histogram of SHBG concentrations in male adolescents**

**S9. Female adolescents. Association between serum concentration of PFOA and proportional Odds ratio for breast development for PFOA concentrations below the median**

**S10. Female adolescents. Association between serum concentration of PFOA and proportional Odds ratio for breast development for PFOA concentrations above the median**

**S11. Female adolescents. Association between serum concentration of PFOA and proportional Odds ratio for breast development for PFOA concentrations below the median on a scale permitting comparison with the graph concerning the association for PFOA concentrations above the median**

**S12. Female adolescents. Association between serum concentration of PFOA and proportional Odds ratio for breast development for PFOA concentrations above the median on a scale permitting comparison with the graph concerning the association for PFHxS concentrations below the median**

**Figures S13. A series of 67 Distance Weighted Least Square Graphs, one for each of the significant associations described by van Larebeke et al. (2025) (ref 1 in this manuscript) for mal adolescents**

**Distance Weighted Least Square Graphs for all associations concerning female adolescents reported as significant in the “original study” by van Larebeke et al.(2025).**

**Fully referenced version of the Discussion**

In our study on the associations of internal exposure to PFAS with thyroid and sex hormone concentrations and pubertal development the strength of the association was, consistent with our work hypothesis, in most cases clearly much stronger below the corresponding median PFAS serum concentration than above. For the sum of PFNA and linear PFHxS, PFOS, and PFOA the median serum concentration for adolescents in our study amounted to 4.40µg/L, for PFOS to2.4µg/L and for PFOA to 1.1 µg/L. EFSA has defined a normative health-based guidance value of a tolerable weekly intake (TWI) of 4.4 nanograms per kilogram of body weight per week for the sum of PFNA and linear PFHxS, PFOS, and PFOA (<https://www.efsa.europa.eu/en/news/pfas-food-efsa-assesses-risks-and-sets-tolerable-intake>). This EFSA norm corresponds to 6.9µG/L serum for the sum of PFNA and linear PFHxS, PFOS, and PFOA. The most strict HBMI reference serum concentration values defined by the German Human Biomonitoring Commission are 5 µg/L for PFOS and 2 µg/L for PFOA (HBM commission, 2016; Hölzer et al., 2021)[24].

Detailed data on exposure-response relations concerning PFAS effects on humans are limited but indicate the occurrence of complex non-monotonic exposure-response curves. Yi et al. (2023) [25] found a U-shaped curve between serum PFAS and depression PHQ-9 score. Gui et al. (2023) [26] found a parabolic exposure-response curve between PFOA exposure and risk of type 2 diabetes. Duan et al. (2021) [27], in a case-control study on 252 cases of Type 2 diabetes and 252 controls, found a non-monotonic inverted U -shaped exposure-response relationships for exposures to PFHxS and PFHpA with risk of diabetes type 2.

Although the estimates for associations limited to PFAS values below the median were often quite strong, statistical significance was often not reached. The lack of statistical significance can be explained by the small number of subjects and also by the limited precision of the determination of the PFAS concentrations at low concentrations such as those below the median values. It can be expected that, for technical reasons, the measurement of PFAS compounds in serum at concentrations below the observed median values is fraught with some uncertainties. That the concentrations below the median were measured with less precision than those above the median is also suggested by the fact that the standard errors on and 95% confidence limit widths of the estimates concerning associations with PFAS concentrations below median values were much larger than those concerning PFAS concentrations above median values (see Table 5). Additionally, in a study limited to individuals for whom the sum of the serum concentrations of PFHxS(total), PFOA(total), PFOS(total), PFDA and PFNA was above the corresponding median serum concentration, the correlations were consistently positive and significant in 13 of the 15 correlations examined (see supplementary material Table S3). In contrast, in a study limited to individuals for whom the same sum of PFAS values was below the corresponding median serum concentration, 5 of the 15 correlations examined were negative, 9 of the 10 positive correlations were significant, and 7 of the 10 positive correlations were weaker than the corresponding correlations above the median (see supplementary Table S3). The fact that the correlation between different PFAS compounds is weaker at concentrations below the median than above the median (see supplementary material Table S3) also points to less precise measurements below the median serum concentrations. In studies seeking to establish a dose-response relationship, inaccuracies in determining exposure will systematically lead to an underestimation of the increase in risk [28]. The lack of precision in the PFAS determinations below the median serum concentrations may contribute to an underestimation of the actual differences in exposure response below and above the median and contributes, together with the decrease in number of observations, to the fact that, in spite of often strong estimates, far less significant associations were found at PFAS concentrations below the median than in the “original study”.

That lower PFAS serum concentrations have per unit of dose (µg/L) stronger effects on certain biological or health parameters can be explained by and is probably due to the fact that these effects are induced by the interaction of PFAS with receptors. Our present finding shares some characteristics with dose -response curves that are quite frequently observed for some other endocrine disrupting chemicals showing stronger associations at lower doses than at higher doses. The mechanisms through which PFAS act include binding to different receptors comprising estrogen receptors [11], vitamin D receptor [29], peroxisome proliferator- activated receptor-alpha [30], constitutive androstane receptor [31] and pregnane X receptor [32]. PFAS thus act as receptor binding endocrine disrupting substances. Natural hormones and receptor-binding endocrine disrupting chemicals such as TCDD tend to display, in arithmetical plots ,in relation with primary effects such as changes in gene expression, an asymptotic-like dose-response curve with an almost linear steep response at very low doses, followed by saturation at higher concentrations reflected in dose-effect curves which are almost horizontal [19]. TCDD has many biological and health effects, most of which are based on the interaction with the AH receptor (AhR), and primary responses, such as the induction of the expression of CYP I A1 and IA2 show a asymptotic-like dose response curve [33]. More complex effects can show different dose-response curves as other signal transduction pathways and tissue- and cell-specific factors can modulate the qualitative and quantitative relationship between receptor occupancy and response [33]. But dose-response curves for effects associated with dioxin exposure often show stronger association (stronger estimate) at low doses than at higher doses. This has been observe for three non-sparsely occurring reproductive outcomes, including not live born, miscarriage, and preterm in a study on male Air Force veterans of the Vietnam War [34]. Also, in a National Health and Nutrition Examination Survey (NHANES) study on 2,992 persons’ a positive linear exposure-effect relation was observed between the log10 transformed serum concentration of 1,2,3,4,6,7,8-heptachlorodibenzofuran and the increase in risk of cancer mortality, implying that per pg/ g blood fat the increase in cancer risk was higher at low exposures than at high exposures [35].

Also for Bisphenol A, one of the most studied endocrine disrupting chemicals, low doses were observed to cause more intense effects than higher doses: the variation of ductal thickness in the mammary gland of rats, indicating an increase in budding, increased between 0 and 25 μg/kg body weight /day, then dropped, with a breaking point between 25 and 250 μg/kg body weight /day, to increase again at doses between 250 and 25000 μg/kg body weight /day [36]. In Cancer tests on the same cohort of rats, Bisphenol A induced a statistically significant increased incidence of adenocarcinoma, increase that was not observed at higher doses [37].Interestingly, Villar-Pazos et al. (2017) [38] showed that BPA dose-response curves can be monotonic for some end points and non-monotonic for other endpoints.

That endocrine disrupting PFAS effects might be, per µg/L serum of internal exposure, relatively more pronounced at low internal exposures than at high internal exposures is consistent with the observation that occupational or severe environmental exposures up to more than a hundred times higher than the environmental exposures observed in general populations, are associated with health effects that are certainly serious [39–42] but not a hundred times worse than those observed in general populations.

Probably the endocrine disrupting effects such as those observed in our studies are due to interactions with receptors as these have been documented in many publications However, PFAS interfere with many other biological mechanisms. They cause oxidative stresss [43,44], infiltrate lipid membranes [45], adhere to proteins [46], affect DNA methylation [47–50], affect acetylation of histones [51] , inhibit of gap junctional intercellular communication [52,53] and increase telomere length [54–56]. So it is likely that they might have other biological and health effects with dose-effect relationships different from those discussed here.

The limitations acknowledged in the “original study” also affect the present study. These concern the number of subjects, the cross-sectional nature, the choice not to adjust for dietary factors, the lack of information about the iodine status of the participants, the use of single-pollutant models not taking into account mixture effects, the assessment of stage of pubertal development resting on self-administered questionnaires. These limitations were discussed in the “original study”. In addition the statistical method to account for the clustering within households (there were 43 households with 2 adolescents participating in the study on the total of 303 adolescents originally recruited for the study) was not available for the present study.

The strengths of the “original study” concerning validated chemical analysis, validated clinical biological analysis and sampling and biological measurements performed by trained study nurses also sustain the present study. Many statistical associations were tested, increasing the likelihood of chance findings. However, in accordance with the views of the epidemiologist Kenneth Rothman [57], and in view of the known endocrine disrupting properties of PFAS—which render observed exposure–effect relations biologically plausible— we did not apply corrections for multiple testing. The way in which testing for statistical significance is done and interpreted can, in some circumstances, prevent important biological/health data or mechanisms from being observed as explained by Kenneth Rothman in a book [58] and in a later publication [59] and by McShane & Gal (2017) [60]

**References**

11. Omoike OE, Pack RP, Mamudu HM, Liu Y, Wang L. A cross-sectional study of the association between perfluorinated chemical exposure and cancers related to deregulation of estrogen receptors. Environ Res. 2021;196:110329. https://doi.org/10.1016/j.envres.2020.110329

19. Lucier GW, Portier CJ, Gallo MA. Receptor mechanisms and dose-response models for the effects of dioxins. Environ Health Perspect. 1993;101:36–44. https://doi.org/10.1289/ehp.9310136

24. Hölzer J, Lilienthal H, Schümann M. Human Biomonitoring (HBM)-I values for perfluorooctanoic acid (PFOA) and perfluorooctane sulfonic acid (PFOS) - Description, derivation and discussion. Regulatory Toxicology and Pharmacology. 2021;121:104862.

25. Yi W, Xuan L, Zakaly HMH, Markovic V, Miszczyk J, Guan H, et al. Association between per- and polyfluoroalkyl substances (PFAS) and depression in U.S. adults: A cross-sectional study of NHANES from 2005 to 2018. Environ Res. 2023;238:117188. https://doi.org/10.1016/j.envres.2023.117188

26. Gui S-Y, Qiao J-C, Xu K-X, Li Z-L, Chen Y-N, Wu K-J, et al. Association between per- and polyfluoroalkyl substances exposure and risk of diabetes: a systematic review and meta-analysis. J Expo Sci Environ Epidemiol. 2023;33:40–55. https://doi.org/10.1038/s41370-022-00464-3

27. Duan Y, Sun H, Yao Y, Li Y, Meng Y, Lu Y, et al. Serum concentrations of per-/polyfluoroalkyl substances and risk of type 2 diabetes: A case-control study. Science of The Total Environment. 2021;787:147476. https://doi.org/10.1016/j.scitotenv.2021.147476

28. Freudenheim JL, Marshall JR. The problem of profound mismeasurement and the power of epidemiological studies of diet and cancer. Nutr Cancer. 1988;11:243–50. https://doi.org/10.1080/01635588809513994

29. Di Nisio A, Rocca MS, De Toni L, Sabovic I, Guidolin D, Dall’Acqua S, et al. Endocrine disruption of vitamin D activity by perfluoro-octanoic acid (PFOA). Sci Rep. 2020;10:16789. https://doi.org/10.1038/s41598-020-74026-8

30. Su S, Billy LJ, Chang S, Gonzalez FJ, Patterson AD, Peters JM. The role of mouse and human peroxisome proliferator-activated receptor-α in modulating the hepatic effects of perfluorooctane sulfonate in mice. Toxicology. 2022;465:153056. https://doi.org/10.1016/j.tox.2021.153056

31. Pierozan P, Cattani D, Karlsson O. Tumorigenic activity of alternative per- and polyfluoroalkyl substances (PFAS): Mechanistic in vitro studies. Science of The Total Environment. 2022;808:151945. https://doi.org/10.1016/j.scitotenv.2021.151945

32. Lai TT, Eken Y, Wilson AK. Binding of Per- and Polyfluoroalkyl Substances to the Human Pregnane X Receptor. Environ Sci Technol. 2020;54:15986–95. https://doi.org/10.1021/acs.est.0c04651

33. SEWALL C. Receptor-mediated events and the evaluation of the Environmental Protection Agency (EPA) of dioxin risks*1. Mutation Research/Fundamental and Molecular Mechanisms of Mutagenesis. 1995;333:111–22. https://doi.org/10.1016/0027-5107(95)00137-9

34. Knafl GJ. An analysis of reproductive outcomes for conceptions of participants of the Air Force Health Study. Reproductive Toxicology. 2023;119:108413. https://doi.org/10.1016/j.reprotox.2023.108413

35. Zheng L, Zhang X, Gao Z, Zhong C, Qiu D, Yan Q. The association between polychlorinated dibenzo-p-dioxin exposure and cancer mortality in the general population: a cohort study. Front Public Health. 2024;12. https://doi.org/10.3389/fpubh.2024.1354149

36. Montévil M, Acevedo N, Schaeberle CM, Bharadwaj M, Fenton SE, Soto AM. A Combined Morphometric and Statistical Approach to Assess Nonmonotonicity in the Developing Mammary Gland of Rats in the CLARITY-BPA Study. Environ Health Perspect. 2020;128. https://doi.org/10.1289/EHP6301

37. NTP Research Report on the CLARITY-BPA Core Study: A Perinatal and Chronic Extended-Dose-Range Study of Bisphenol A in Rats. 111 TW Alexander Dr, Durham, NC 27709; 2018 Sep. https://doi.org/10.22427/NTP-RR-9

38. Villar-Pazos S, Martinez-Pinna J, Castellano-Muñoz M, Alonso-Magdalena P, Marroqui L, Quesada I, et al. Molecular mechanisms involved in the non-monotonic effect of bisphenol-a on Ca2+ entry in mouse pancreatic β-cells. Sci Rep. 2017;7:11770. https://doi.org/10.1038/s41598-017-11995-3

39. Porter AK, Kleinschmidt SE, Andres KL, Reusch CN, Krisko RM, Taiwo OA, et al. Occurrence of COVID-19 and serum per- and polyfluoroalkyl substances: A case-control study among workers with a wide range of exposures. Glob Epidemiol. 2024;7:100137. https://doi.org/10.1016/j.gloepi.2024.100137

40. Andres KL, Olsen GW, Krisko RM, Nunnally MC, Boeding RR, Leniek KL, et al. An investigation of 3M Cordova, IL production worker’s per- and polyfluoroalkyl substances biomonitoring results and mortality experience. Int J Hyg Environ Health. 2024;256:114321. https://doi.org/10.1016/j.ijheh.2024.114321

41. Batzella E, Girardi P, Russo F, Pitter G, Da Re F, Fletcher T, et al. Perfluoroalkyl substance mixtures and cardio-metabolic outcomes in highly exposed male workers in the Veneto Region: A mixture-based approach. Environ Res. 2022;212:113225. https://doi.org/10.1016/j.envres.2022.113225

42. Vieira VM, Hoffman K, Shin H-M, Weinberg JM, Webster TF, Fletcher T. Perfluorooctanoic Acid Exposure and Cancer Outcomes in a Contaminated Community: A Geographic Analysis. Environ Health Perspect. 2013;121:318–23. https://doi.org/10.1289/ehp.1205829

43. Siwakoti RC, Park S, Ferguson KK, Hao W, Cantonwine DE, Mukherjee B, et al. Prenatal per- and polyfluoroalkyl substances (PFAS) and maternal oxidative stress: Evidence from the LIFECODES study. Chemosphere. 2024;360:142363. https://doi.org/10.1016/j.chemosphere.2024.142363

44. Widhalm R, Granitzer S, Natha B, Zoboli O, Derx J, Zeisler H, et al. Perfluorodecanoic acid (PFDA) increases oxidative stress through inhibition of mitochondrial β-oxidation. Environmental Pollution. 2025;367:125595. https://doi.org/10.1016/j.envpol.2024.125595

45. Nouhi S, Ahrens L, Campos Pereira H, Hughes A V., Campana M, Gutfreund P, et al. Interactions of perfluoroalkyl substances with a phospholipid bilayer studied by neutron reflectometry. J Colloid Interface Sci. 2018;511:474–81. https://doi.org/10.1016/j.jcis.2017.09.102

46. Fedorenko M, Alesio J, Fedorenko A, Slitt A, Bothun GD. Dominant entropic binding of perfluoroalkyl substances (PFASs) to albumin protein revealed by 19F NMR. Chemosphere. 2021;263:128083. https://doi.org/10.1016/j.chemosphere.2020.128083

47. Leung Y-K, Ouyang B, Niu L, Xie C, Ying J, Medvedovic M, et al. Identification of sex-specific DNA methylation changes driven by specific chemicals in cord blood in a Faroese birth cohort. Epigenetics. 2018;13:290–300. https://doi.org/10.1080/15592294.2018.1445901

48. Quaid M, Goodrich JM, Calkins MM, Graber JM, Urwin D, Gabriel J, et al. Firefighting, per‐ and polyfluoroalkyl substances, and DNA methylation of genes associated with prostate cancer risk. Environ Mol Mutagen. 2024;65:55–66. https://doi.org/10.1002/em.22589

49. Goodrich JM, Calkins MM, Caban-Martinez AJ, Stueckle T, Grant C, Calafat AM, et al. Per- and Polyfluoroalkyl Substances, Epigenetic Age and DNA Methylation: A Cross-Sectional Study of Firefighters. Epigenomics. 2021;13:1619–36. https://doi.org/10.2217/epi-2021-0225

50. Xu Y, Jurkovic-Mlakar S, Lindh CH, Scott K, Fletcher T, Jakobsson K, et al. Associations between serum concentrations of perfluoroalkyl substances and DNA methylation in women exposed through drinking water: A pilot study in Ronneby, Sweden. Environ Int. 2020;145:106148. https://doi.org/10.1016/j.envint.2020.106148

51. Alam MN, Han X, Nan B, Liu L, Tian M, Shen H, et al. Chronic low-level perfluorooctane sulfonate (PFOS) exposure promotes testicular steroidogenesis through enhanced histone acetylation. Environmental Pollution. 2021;284:117518. https://doi.org/10.1016/j.envpol.2021.117518

52. Hu W. Inhibition of Gap Junctional Intercellular Communication by Perfluorinated Compounds in Rat Liver and Dolphin Kidney Epithelial Cell Lines in Vitro and Sprague-Dawley Rats in Vivo. Toxicological Sciences. 2002;68:429–36. https://doi.org/10.1093/toxsci/68.2.429

53. López‐Arellano P, López‐Arellano K, Luna J, Flores D, Jiménez‐Salazar J, Gavia G, et al. Perfluorooctanoic acid disrupts gap junction intercellular communication and induces reactive oxygen species formation and apoptosis in mouse ovaries. Environ Toxicol. 2019;34:92–8. https://doi.org/10.1002/tox.22661

54. Clarity C, Trowbridge J, Gerona R, Ona K, McMaster M, Bessonneau V, et al. Associations between polyfluoroalkyl substance and organophosphate flame retardant exposures and telomere length in a cohort of women firefighters and office workers in San Francisco. Environmental Health. 2021;20:97. https://doi.org/10.1186/s12940-021-00778-z

55. Eick SM, Goin DE, Cushing L, DeMicco E, Park J-S, Wang Y, et al. Mixture effects of prenatal exposure to per- and polyfluoroalkyl substances and polybrominated diphenyl ethers on maternal and newborn telomere length. Environmental Health. 2021;20:76. https://doi.org/10.1186/s12940-021-00765-4

56. Huang H, Wang Q, He X, Wu Y, Xu C. Association between polyfluoroalkyl chemical concentrations and leucocyte telomere length in US adults. Science of The Total Environment. 2019;653:547–53. https://doi.org/10.1016/j.scitotenv.2018.10.400

57. Rothman Kenneth J. Modern epidemiology. Boston: Little Brown And Company  ISBN 0-316-75776-4. ; 1986.

58. Rothman Kenneth J. Modern epidemiology. Boston: Little Brown And Company ISBN 0-316-75776-4; 1986.

59. Rothman KJ. Rothman Responds to “Surprise!” Am J Epidemiol. 2021;190:194–5. https://doi.org/10.1093/aje/kwaa137

60. McShane BB, Gal D. Statistical Significance and the Dichotomization of Evidence. J Am Stat Assoc. 2017;112:885–95. https://doi.org/10.1080/01621459.2017.1289846
